# Expression of Nestin associates with *BRCA1* mutations, a basal-like phenotype and aggressive breast cancer

**DOI:** 10.1038/s41598-017-00862-w

**Published:** 2017-04-24

**Authors:** Kristi Krüger, Elisabeth Wik, Gøril Knutsvik, Hawa Nalwoga, Tor A. Klingen, Jarle B. Arnes, Ying Chen, Monica Mannelqvist, Konstantina Dimitrakopoulou, Ingunn M. Stefansson, Even Birkeland, Turid Aas, Nicholas P. Tobin, Inge Jonassen, Jonas Bergh, William D. Foulkes, Lars A. Akslen

**Affiliations:** 1 Centre for Cancer Biomarkers CCBIO, Department of Clinical Medicine, Section for Pathology, University of Bergen, Bergen, Norway; 2 Department of Pathology, Haukeland University Hospital, Bergen, Norway; 3 Department of Pathology, Makerere University College of Health Sciences, P. O. Box 7072 Kampala, Uganda; 4 Department of Pathology, Vestfold Hospital, Tønsberg, Norway; 5 Department of Pathology, Akershus University Hospital, Lørenskog, Norway; 6 Centre for Cancer Biomarkers CCBIO and Computational Biology Unit, Department of Informatics, University of, Bergen, Norway; 7 Department of Oncology and Pathology, Karolinska Institute and University Hospital, Stockholm, Sweden; 8 Department of Surgery, Haukeland University Hospital, Bergen, Norway; 9 Program in Cancer Genetics, Departments of Oncology and Human Genetics, McGill University, 546 Pine Avenue West, Montreal, QC H2W 1S6 Canada

## Abstract

We here examined whether Nestin, by protein and mRNA levels, could be a predictor of *BRCA1* related breast cancer, a basal-like phenotype, and aggressive tumours. Immunohistochemical staining of Nestin was done in independent breast cancer hospital cohorts (Series I-V, total 1257 cases). Also, TCGA proteomic data (n = 103), mRNA microarray data from TCGA (n = 520), METABRIC (n = 1992), and 6 open access breast cancer datasets (n = 1908) were analysed. Patients with Nestin protein expression in tumour cells more often had *BRCA1* germline mutations (OR 8.7, p < 0.0005, Series III), especially among younger patients (<40 years at diagnosis) (OR 16.5, p = 0.003). Nestin protein positivity, observed in 9–28% of our hospital cases (Series I-IV), was independently associated with reduced breast cancer specific survival (HR = 2.0, p = 0.035) and was consistently related to basal-like differentiation (by Cytokeratin 5, OR 8.7–13.8, p < 0.0005; P-cadherin OR 7.0–8.9, p < 0.0005; EGFR staining, OR 3.7–8.2, p ≤ 0.05). Nestin mRNA correlated significantly with Nestin protein expression (ρ = 0.6, p < 0.0005), and high levels were seen in the basal-like intrinsic subtype. Gene expression signalling pathways linked to high Nestin were explored, and revealed associations with stem-like tumour features. In summary, Nestin was strongly associated with germline *BRCA1* related breast cancer, a basal-like phenotype, reduced survival, and stemness characteristics.

## Introduction

Breast cancers are diverse with respect to morphological features and biological characteristics. Based on gene expression patterns, Perou *et al*. identified five intrinsic subtypes: luminal A and B, basal-like, HER2 (human epidermal growth factor receptor 2) enriched and normal breast-like subgroups. Approximately 15% of breast cancers are of the basal-like phenotype, and these are associated with early onset, high tumour grade, and reduced survival^1, 2^. Further, basal-like breast cancer is associated with *BRCA1* germline mutations^3^, and is more prevalent in certain populations^1^. Most of the basal-like tumours lack traditional treatment targets. For practical reasons, surrogate markers studied on tissue specimens are often used to define the basal-like subtype, instead of the more time-consuming and costly gene expression profiling^4^. However, there is currently no consensus on the preferred definition of basal-like tumours, although several individual biomarkers and combinations have been suggested^5–7^.

Nestin is an intermediate filament protein originally reported as a neuronal stem-cell or progenitor marker^8^. It was later observed in non-neural tissues, including myoepithelial mammary cells^9^ and immature blood vessels^10^. Importantly, Nestin expression has been reported in pathological conditions such as tissue injury^11^ and malignant tumours^12^. Its expression correlates with the degree of malignancy^13^, and Nestin has been promoted as a cancer stem-cell marker^14^.

In breast cancer, Nestin has been associated with a basal-like differentiation in small studies by us and others^5, 9, 15^. Here, the main objective was to evaluate Nestin expression in larger breast cancer series and to explore whether this marker can predict *BRCA1* associated cancers. Also, associations with various basal-like profiles and subgroups were explored, along with features of aggressive tumour behaviour. Finally, signalling pathways linked to Nestin expression and potential stemness markers were examined. Different cohorts were studied to validate findings across populations, and over 5600 patients were included for protein or gene expression analyses.

## Results

### Nestin expression is associated with *BRCA1* germline mutations

**Figure 1:**
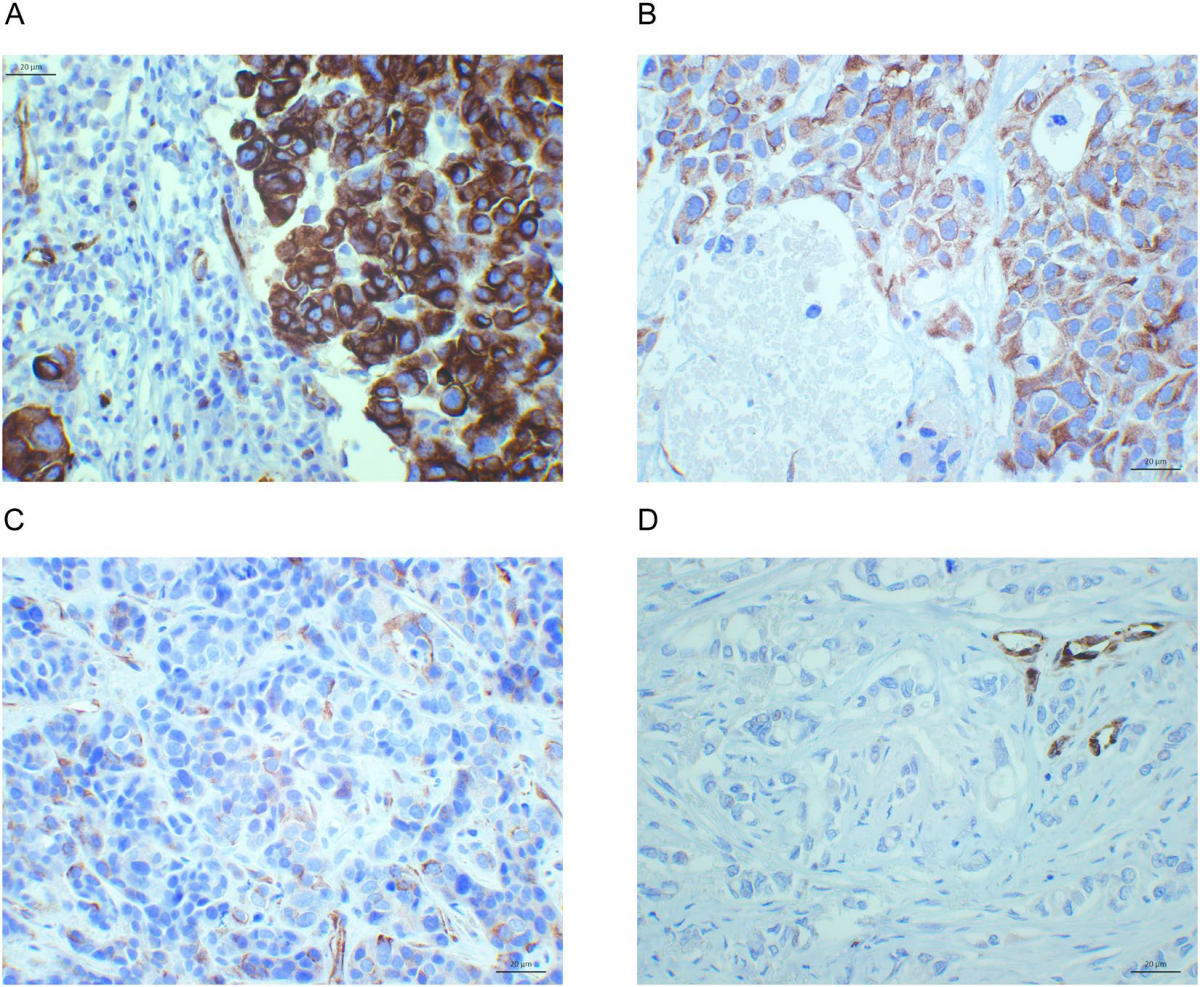
Nestin protein expression by immunohistochemistry. Nestin protein expression, strong (**A**), moderate (**B**), weak (**C**) and negative (**D**), in breast cancer tumour cells, with some positive tumour vessels in D (40﻿0x).

Nestin protein expression in breast cancer cells was found by immunohistochemistry in 9%, 13%, 28% and 24% of the cases in Series I-IV, respectively (Fig. 1). *BRCA1* positive cases more often had Nestin positive tumours, compared with cases without *BRCA1* germline mutations, OR (odds ratio) 8.7 (p < 0.0005, Series III) (Table 1), with a sensitivity of 62% and specificity of 84%. In this series enriched for *BRCA1* germline mutations, the positive and negative predictive values for Nestin with respect to *BRCA1* germline mutations were 65% and 82%, respectively.

**Table 1:** Nestin protein expression and *BRCA1* germline mutation status (Series III).

*BRCA1*	Nestin−n (%)	Nestin+n (%)	OR (95% CI)
Total			
Negative	79 (84.0)	15 (16.0)	1.0
Positive	17 (37.8)	28 (62.2)	8.7 (3.8–19.6)***
<40 years			
Negative	12 (75.0)	4 (25.0)	1.0
Positive	2 (15.4)	11 (84.6)	16.5 (2.5–109)**
≥40 years			
Negative	66 (85.7)	11 (14.3)	1.0
Positive	15 (50.0)	15 (50.0)	6.0 (2.3–15.7)***

n: number of patients; OR: odds ratio; CI: confidence interval; P-values by Pearson’s chi-square test, *<0.05, **<0.01, ***<0.001. *BRCA2* germline mutated patients are excluded from the analysis. Missing data: Age: n = 3.

The association between Nestin and *BRCA1* germline mutations was stronger among patients under 40 years, OR 16.5, than among those 40 years or above, OR 6.0 (Table 1). When including Nestin in addition to other basal markers, CK5 (Cytokeratin 5), EGFR (epidermal growth factor receptor), and P-cadherin, in a multiple logistic regression analysis of *BRCA1* status, only Nestin and P-cadherin significantly predicted *BRCA1* germline mutations, and Nestin was the strongest predictor (Nestin OR 5.4, p < 0.0005) (Table 2).

**Table 2:** Prediction of *BRCA1* germline mutation (n = 45) by multivariate logistic regression (Series III).

Variables	n (%)	OR (95% CI)	*P*
Nestin			
Negative	95 (68.8)	1.0	<0.0005
Positive	43 (31.2)	5.4 (2.2–13.3)	
CK5			
Negative	104 (75.4)	1.0	NS
Positive	34 (24.6)	1.2 (0.4–3.7)	
EGFR			
Negative	116 (84.1)	1.0	NS
Positive	22 (15.9)	1.2 (0.4–3.8)	
P-cadherin			
Negative	68 (49.3)	1.0	0.019
Positive	70 (50.7)	3.1 (1.2–7.9)	

n: number of patients; OR: odds ratio; CI: confidence interval; P: p-values. CK: cytokeratin; EGFR: epidermal growth factor receptor. Cut-off values: CK5 positive: SI(staining index)>0; EGFR positive: >1%, Dako criteria; P-cadherin positive: SI > 3. *BRCA2* germline mutated patients were excluded from the analysis. Missing data: EGFR: n = 1. Only cases with information on all variables were included (n = 138).

When also including the triple negative profile (TNP) in the multivariate logistic regression analysis, prediction of *BRCA1* status was significant for TNP (p < 0.0005), borderline for Nestin (p = 0.083), and not significant for CK5, EGFR, and P-cadherin. In younger patients (<40 years), Nestin was the only significant predictor of *BRCA1* status (p = 0.048), whereas TNP, CK5, EGFR, and P-cadherin, were not significant (Table 3). If CK5 is substituted by Nestin in the core basal profile^7^ (ER− HER2− EGFR+ and/or Nestin+), and compared with the TNP, both profiles significantly predicted *BRCA1* germline mutations (p = 0.018 and p < 0.0005, respectively).

**Table 3:** Prediction of *BRCA1* germline mutation (n = 13) by multivariate logistic regression in patients below 40 years (Series III).

	n (%)	OR (95% CI)	*P*
Nestin			
Negative	14 (48.3)	1.0	0.048
Positive	15 (51.7)	12.9 (1.0–164)	
CK5			
Negative	19 (65.5)	1.0	NS
Positive	10 (34.4)	0.8 (0.1–8.2)	
EGFR			
Negative	21 (72.4)	1.0	NS
Positive	8 (27.6)	1.7 (0.1–22.2)	
P-cadherin			
Negative	11 (37.9)	1.0	NS
Positive	18 (62.1)	0.2 (0.0–6.2)	
TNP			
Absent	15 (51.7)	1.0	NS
Present	14 (48.3)	6.0 (0.4–85.7)	

n: number of patients; OR: odds ratio; CI: confidence interval; P: p-values. CK: cytokeratin; EGFR: epidermal growth factor receptor; TNP: triple negative profile. Cut-off values: CK5 positive: SI (staining index)> 0; EGFR positive: >1%, Dako criteria; P-cadherin positive: SI > 3. *BRCA2* germline mutated patients were excluded from the analysis. Only cases with information on all variables were included (n = 29).

In the TCGA (The Cancer Genome Atlas) dataset, high Nestin mRNA (*NES*) and high Nestin signature score (by upper quartile) were associated with *BRCA1* germline mutations (OR 5.5 and 14.4, respectively) (Table 4). Similar findings were observed in two *BRCA*-validation datasets (GSE25307 and GSE40115, OR range 2.3 to 10.2 for Nestin mRNA and signature score). Also, Nestin mRNA and signature score significantly predicted *BRCA1* germline mutation status when analysed as continuous variables in all three gene expression cohorts (not shown). The Nestin mRNA signature score was the only significant predictor of *BRCA1* germline mutations (OR 1.03, p = 0.001) when included together with EGFR, CK5, and P-cadherin by multiple logistic regression.

**Table 4:** *BRCA1* germline mutation by Nestin mRNA expression and Nestin mRNA signature score (microarray data).

Germline *BRCA1*			
	Negative n (%)	Positive n (%)	OR (95% CI)
**TCGA**			
Nestin mRNA			
Low	360 (75.9)	4 (36.4)	1.0
High	114 (24.1)	7 (63.6)	5.5 (1.6–19.2)^a,^**
Nestin signature score			
Low	361 (76.2)	2 (18.2)	1.0
High	113 (23.8)	9 (81.8)	14.4 (3.1–67.5)^a,^***
**GSE40115**			
Nestin mRNA			
Low	100 (78.1)	20 (60.6)	1.0
High	28 (21.9)	13 (39.4)	2.3 (1.0–5.2)*
Nestin signature score			
Low	107 (83.6)	11 (33.3)	1.0
High	21 (16.4)	22 (66.7)	10.2 (4.3–24.1)***
**GSE25307**			
Nestin mRNA			
Low	366 (78.2)	17 (50.0)	1.0
High	102 (21.8)	17 (50.0)	3.6 (1.8–7.3)***

^a^Fisher’s exact test. n: number of patients; OR: odds ratio; CI: confidence interval; P-values by Pearson’s chi-square test, *<0.05, **<0.01, ***<0.001. *BRCA2* germline mutated patients are excluded from the analysis. Cut-off value: high: ≥upper quartile. Missing data: *BRCA*-status: TCGA: n = 23, GSE25307: n = 36.

When including TNP and the basal-like subgroup (by PAM50), only Nestin mRNA signature score significantly predicted *BRCA1* germline mutation status (TCGA dataset, OR 1.03, p = 0.044), whereas the TNP, basal-like subgroup, CK5, EGFR, and P-cadherin did not.

In the TCGA proteomic dataset (n = 103), all 3 patients with *BRCA1* germline mutations had high Nestin protein signature score (p = 0.015). Neither Nestin protein expression, high Nestin mRNA nor high signature score were associated with *BRCA2* germline mutations in these series (data not shown).

### Nestin expression is associated with a basal-like phenotype

Nestin protein positivity was significantly associated with hormone receptor negativity and high proliferation by Ki-67 or mitosis count (p ≤ 0.001) (Table 5). Nestin expression was associated with basal-like differentiation in Series I-IV, by CK5, OR 8.7–13.8 (p < 0.0005), and P-cadherin, OR 7.0–8.9 (p < 0.0005) (Supplementary Table S1). Also, Nestin was significantly associated with EGFR, OR 3.7–8.2 (p ≤ 0.05), p53 expression, OR 2.5–8.5 (p ≤ 0.007), and with the core basal phenotype^7^ (CBP; ER− HER2− CK5+ and/or EGFR+, OR 17.4–27.0, p < 0.0005). The positive predictive values of Nestin detecting the core basal phenotype were 58%, 57%, and 65% and the negative predictive values were 95%, 93%, and 92% in Series I, III, and IV, respectively.

**Table 5:** Nestin protein expression by receptor status and tumour cell proliferation.

Variables	Series I (n = 528)		Series II (n = 279)		Series III (n = 181)		Series IV (n = 187)	
	Nestin +n (%)	OR (95% CI)	Nestin +n (%)	OR (95% CI)	Nestin + n (%)	OR (95% CI) n (%)	Nestin +n (%)	OR (95% CI)
ER								
Positive	18 (4.0)	1.0	18 (7.4)	1.0	5 (5.5)	1.0	6 (8.3)	1.0
Negative	32 (38.6)	14.9 (7.8–28.4)***	17 (48.6)	11.9 (5.2–26.9)^a,^***	46 (51.1)	18.0 (6.7–48.5)***	38 (33.6)	5.6 (2.2–14.0)***
PR								
Positive	13 (3.5)	1.0	14 (7.7)	1.0	10 (11.1)	1.0	2 (3.8)	1.0
Negative	37 (23.6)	8.5 (4.4–16.5)***	21 (21.9)	3.4 (1.6–7.0)**	41 (45.1)	6.6 (3.0–14.3)***	42 (31.8)	11.9 (2.8–51.2)***
HER2								
Negative	40 (8.8)	1.0	32 (12.7)	1.0	50 (30.1)	1.0	43 (27.9)	1.0
Positive	10 (14.1)	1.7 (0.8–3.6)	3 (11.1)	0.9 (0.2–3.0)^a^	1 (6.7)	0.2 (0.0–1.3)^a^	1 (3.2)	0.1 (0.0–0.7)**
Ki-67^b^								
Low	13 (3.3)	1.0	11 (5.3)	1.0	21 (16.9)	1.0	23 (16.5)	1.0
High	37 (28.0)	11.4 (5.9–22.4)***	24 (33.8)	9.1 (4.2–20.0)***	27 (60.0)	7.4 (3.4–15.7)***	21 (44.7)	4.1 (2.0–8.4)***

^a^Fisher’s exact test. ^b^Series III, mitosis count by upper quartile, because data on Ki-67 was not available. n: number of patients; OR: odds ratio; CI: confidence interval; p-values by Pearson’s chi-square test, *<0.05, **<0.01, ***<0.001; ER: oestrogen receptor; PR: progesterone receptor; HER2: human epidermal growth factor receptor 2. Cut-off values: ER and PR positive: ≥10%; HER2: see Supplementary Methods; Ki-67: series specific upper quartile, Series I: 31.5, Series II: 23.0, Series IV: 38.7%. Missing data: Series I: HER2: n = 1, Ki-67: n = 1, Series III: Mitosis: n = 12, Series IV: ER: n = 2, PR: n = 2, HER2: n = 2, Ki-67: n = 1.

Five immunohistochemistry-based basal-like profiles were all strongly and significantly associated with Nestin expression (OR 9.4–35.5, p ≤ 0.005). Overall, the sensitivity for detecting basal-like cases by Nestin expression was 50–79%, and the specificity was 77–93% (Supplementary Table S1).

**Figure 2:**
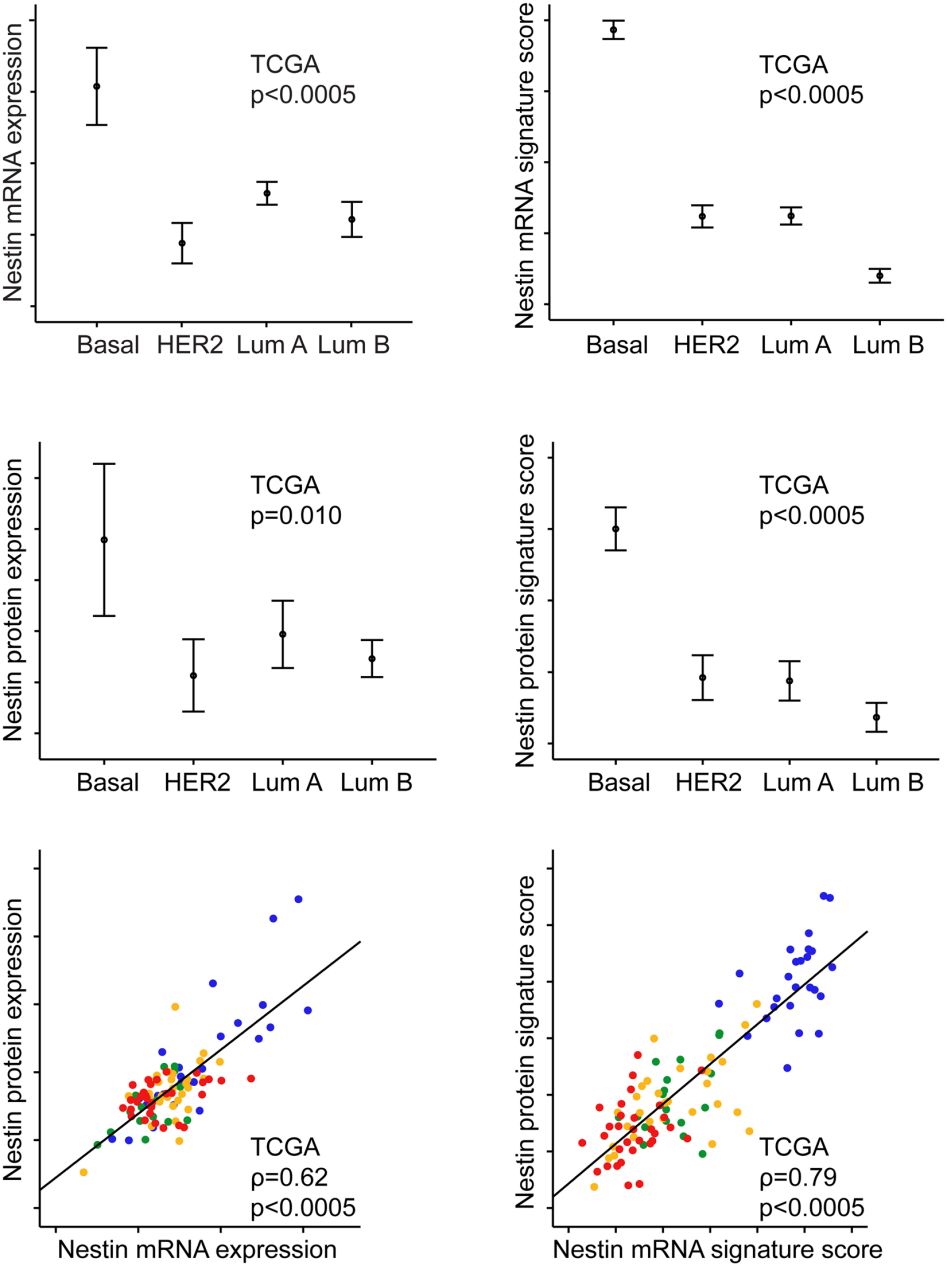
Nestin mRNA, protein expression and signature scores across molecular subtypes of breast cancer in the TCGA dataset, and correlation of Nestin mRNA, protein expression and signature scores. TCGA microarray data (n = 505) of Nestin mRNA (*NES*) expression, Nestin mRNA signature score (44 genes), proteomic data (n = 103) of Nestin protein expression and Nestin protein signature score (27 of 44 proteins) across molecular subtypes of breast cancer. Data is presented by error-bars with 95% confidence interval of the mean, and p-values by the Kruskal-Wallis test. Scatter plots for Nestin mRNA and protein expression and mRNA and protein signature score with p-values by Spearman’s rank correlation and the coefficients (ρ) are presented. The normal breast-like category was excluded. Molecular subtypes are indicated with colours; basal-like: blue; HER2 enriched: green; luminal A: orange; luminal B; red.

In the TCGA dataset, Nestin protein expression and a Nestin protein signature score were correlated to Nestin mRNA levels (ρ = 0.6, p < 0.0005 for mRNA and protein correlation, and ρ = 0.8, p < 0.0005 for mRNA signature score and protein signature score correlation) (Fig. 2). Nestin protein expression and the protein signature score differed significantly across molecular subtypes (p ≤ 0.010), being highest in basal-like cases. Nestin mRNA and the mRNA signature score also consistently showed higher expression in basal-like tumours, compared with HER2 enriched and luminal A and B subtypes (p-values < 0.0005 for most comparisons; for individual p-values, see Fig. 2 and Supplementary Fig. S1A and B). By multiple logistic regression analyses (TCGA dataset), Nestin mRNA expression and Nestin protein expression significantly and independently predicted the basal-like subgroup (by PAM50) when compared with CK5, EGFR, and P-cadherin expression (Nestin mRNA, OR 2.3, p < 0.0005; Nestin protein, OR 10.5, p < 0.0005, Supplementary Table S2). Similar results were obtained when including Nestin mRNA or protein signature scores in the same analysis (mRNA signature score, OR 1.1, p < 0.0005; Nestin protein signature score, OR 1.9, p = 0.003). Also, Nestin mRNA expression predicted the basal-like subgroup in the smaller GEO (Gene Expression Omnibus) datasets (data not shown).

### Nestin expression is associated with aggressive tumour characteristics

Nestin protein expression by immunohistochemistry was consistently associated with higher histological grade (OR 2.7–14.3), larger tumours (OR 1.8–2.4, Series I-III), lymph node negativity (OR 3.3, Series III), and interval-detected compared with screening-detected tumours (OR 1.8–2.7, Series I-II) (Supplementary Table S3). Nestin positive tumours more often showed blood vessel invasion (OR 2.6, Series II), but not lymphatic vessel invasion. In addition, Nestin was associated with selected angiogenesis related markers (Supplementary Table S3).

In cases with distant metastasis (Series V, n = 35), Nestin protein expression was found in 29% of metastases and 31% of paired primary tumours, with no significant difference between the two groups (McNemar’s test, p = 1.0). Five patients (14%) showed discordant Nestin status between the primary tumour and the corresponding metastasis.

### Nestin expression is associated with reduced patient survival

**Figure 3:**
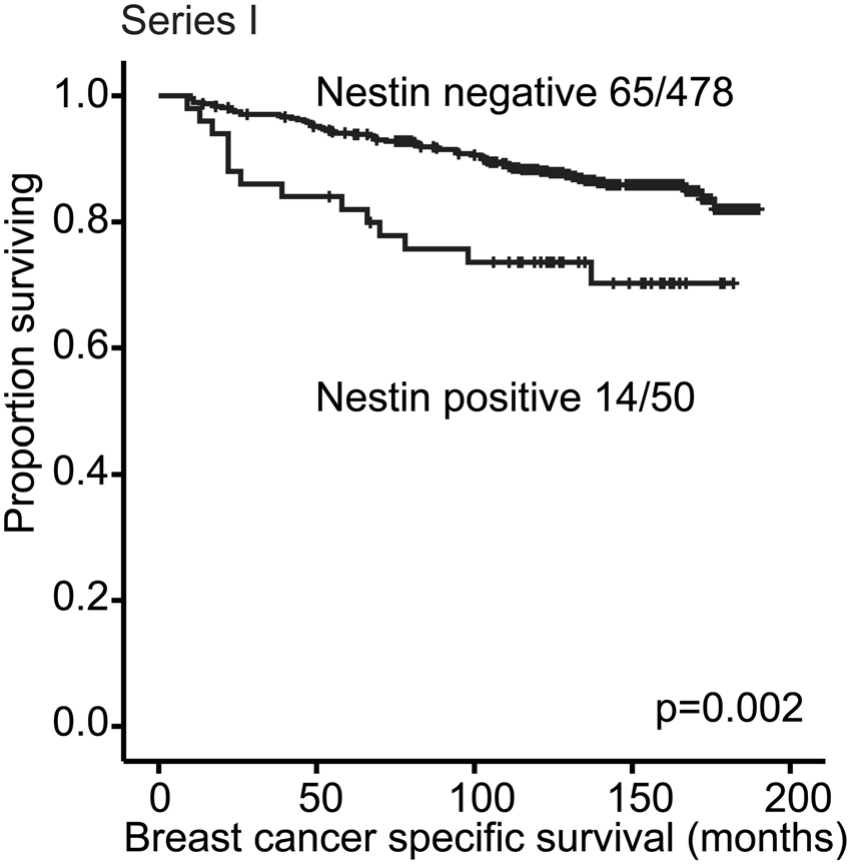
Breast cancer specific survival according to Nestin status. Kaplan-Meier univariate breast cancer specific survival analysis in Series I according to Nestin protein expression (log-rank test for difference). For each category, the number of breast cancer deaths is given, followed by the total number of cases in each category.

Nestin protein expression was associated with reduced breast cancer specific survival (p = 0.002, Series I) (Fig. 3). When including basic clinico-pathological factors, i.e. tumour diameter, histological grade and lymph node status in multivariate analysis, Nestin expression was independently associated with reduced survival (HR, hazard ratio = 2.0, p = 0.035) (Table 6).

**Table 6:** Univariate and multivariate survival analysis (Cox’ proportional hazards regression) with death from breast cancer as end-point (Series I).

Variables	n	Univariate HR (95% CI)	*P*	Multivariate HR (95% CI)	*P*
Tumour diameter					
<2.0 cm	358	1.0	<0.0005	1.0	0.001
≥2.0 cm	165	3.7 (2.3–5.9)		2.3 (1.4–3.7)	
Histological grade					
1–2	433	1.0	<0.0005	1.0	NS
3	90	2.4 (1.5–3.9)		1.5 (0.8–2.5)	
Nodal status					
Negative	382	1.0	<0.0005	1.0	<0.0005
Positive	141	4.7 (3.0–7.4)		3.7 (2.3–6.0)	
Nestin					
Negative	473	1.0	0.002	1.0	0.035
Positive	50	2.5 (1.4–4.5)		2.0 (1.1–3.8)	

n: number of patients; HR: hazard ratio; CI: confidence interval; P: p-values; NS: not significant. Only patients with information on all variables were included in the analysis (n = 523).

The TNP was associated with reduced breast cancer specific survival (p = 0.001). When stratifying for TNP status, Nestin was significantly associated with reduced breast cancer specific survival in univariate survival analysis in the TNP absent group (log-rank test, p = 0.003), but not in the TNP present group (Supplementary Fig. S2). When TNP was included in a multivariate Cox’ proportional hazards model in addition to Nestin and basic clinico-pathological features, as well as a Nestin-TNP interaction term, both Nestin and TNP significantly and independently associated with poorer prognosis (HR = 2.4, p = 0.034 and HR = 3.3, p = 0.004, respectively), and Nestin and TNP tended to interact (p = 0.055).

Among non-basal cases, defined by absence of the ER− HER2− CK5+ profile or ER− HER2− P-cadherin + profile, Nestin was significantly associated with poorer breast cancer specific survival (log-rank test, p = 0.003 and p = 0.004, respectively).

High Nestin mRNA and signature score were significantly associated with reduced recurrence-free survival using the Kaplan-Meier plotter online survival tool (p < 0.0005 and p = 0.0024, respectively) (Supplementary Fig. S3), also when selecting for the basal-like subtype (Nestin mRNA, p = 0.0044, data not shown). In the METABRIC (Molecular Taxonomy of Breast Cancer International Consortium) cohort, patients with high Nestin signature score had a reduced breast cancer specific survival (p = 0.01). For Nestin mRNA expression, the association was also significant (p = 0.037, by median) (data not shown).

### High Nestin mRNA associates with transcriptional alterations reflecting stemness and mesenchymal features

Gene sets reflecting Wnt/β-Catenin signalling and KRAS activation were significantly enriched in Nestin mRNA high cases (TCGA dataset/GSEA, see Supplementary Methods). Wnt/β-Catenin and KRAS signature scores were generated in the TCGA and GEO datasets, and correlated significantly with Nestin mRNA and signature scores (Spearman’s ρ > 0.4, p-values < 0.0005 and ρ > 0.2, p-values < 0.003, respectively, for most comparisons, see Supplementary Fig. S4 for individual ρ- and p-values).

Nestin mRNA and signature score differed across the 6 triple-negative subgroups described by Lehmann *et al*.^16^ (TCGA and GSE25066) (Supplementary Fig. S5). Nestin mRNA and signature score were highest in the basal-like 1, mesenchymal and mesenchymal stem-like subgroups combined, compared with the other subgroups for both datasets (Mann-Whitney U test, p ≤ 0.009).

By TEAK analysis, retinol metabolism was among the most altered pathways between Nestin mRNA high (>upper quartile) and low (<lower quartile) groups. Further, several Wnt-subpathways were within the top 10% of the differential subpathways, supporting GSEA/M SigDB findings.

Nestin mRNA and signature score (TCGA dataset) significantly correlated with the mammary stem cell enriched and the luminal progenitor signature score by Lim *et al*.^17^ (Nestin signature score ρ = 0.70 and ρ = 0.76, respectively), and negatively correlated with the mature luminal signature score (Nestin signature score ρ = −0.66). Weaker correlations were seen with the stromal signature score (Nestin signature score ρ = 0.16) (all p-values < 0.0005; for individual ρ-values, see Supplementary Fig. S6). Similar to findings by Lim *et al*., mammary stem cell enriched and luminal progenitor signature scores differed across molecular subtypes (Kruskal-Wallis test, p < 0.0005), and both were highest in the basal-like subtype, whereas the mature luminal signature was highest in luminal A and B subtypes in this dataset. The luminal progenitor score was higher in *BRCA1* germline mutated patients compared to cases without *BRCA* mutations (Mann-Whitney U test, p < 0.0005); in contrast to the results from Lim *et al*., the mammary stem cell enriched signature score was higher in patients with *BRCA1* germline mutations (Mann-Whitney U test, p = 0.002). The mature luminal signature score was significantly lower in patients with *BRCA1* germline mutations (Mann-Whitney U test, p < 0.0005).

## Discussion

Intrinsic molecular subtypes of breast cancer were described by Perou *et al*.^18^ based on gene expression patterns. Since then, several individual or combined immunohistochemical surrogate markers have been suggested to delineate the basal-like phenotype, but no consensus has been reached^5–7^. As such, Nestin positivity has been associated with basal-like features in small studies, by us and others^5, 9, 15^. Here, Nestin expression in breast cancer has been mapped and validated in multiple cohorts from different populations. Our findings, on protein and mRNA levels, strongly indicate that Nestin is associated with *BRCA1* related breast cancer, the basal-like phenotype, high-grade tumour features and reduced survival with independent significance.

In a study of immunohistochemical markers in comparison with gene expression patterns, Won *et al*.^5^ reported that Nestin had the best combination of sensitivity (54%) and specificity (96%) among positively expressed markers. Nestin was suggested as the best individual marker for basal-like differentiation. Here, Nestin protein expression was found to be superior to other basal-like markers such as CK5, P-cadherin and EGFR by multivariate analysis. Further, Nestin detected the core basal phenotype by a sensitivity of 58–65% and a specificity of 92–95%, similar to findings by Won *et al*.^5^. Very few of the non-basal cases were Nestin positive, whereas some of the basal-like tumours were Nestin negative, supporting that the basal-like phenotype is heterogeneous, an observation that should be further explored^16, 19^. Nestin showed strong associations with increased proliferation by Ki-67 expression and p53 positivity, key characteristics of the basal-like category^20^. Our study strongly supports previous findings, that Nestin expression, as an individual immunohistochemical marker, is useful to identify a basal-like phenotype, and that Nestin might be stronger than other markers in this respect.

Importantly, we found a strong association between Nestin positivity and *BRCA1* germline mutations, especially among younger patients. This has been suggested only once before, in a small series of eight patients^9^. The association between Nestin and *BRCA1* status was supported by analyses of gene and protein expression data (TCGA, GSE40115, and GSE25307 cohorts). Compared with CK5, EGFR, and P-cadherin, protein expression of Nestin protein was the strongest predictor of *BRCA1* germline mutations by multivariate analysis, supported by similar findings for Nestin mRNA and signature score. Compared with the triple negative profile (TNP), CK5, EGFR, and P-cadherin, only Nestin protein expression significantly predicted *BRCA1* germline mutation status in patients under 40 years. Also, the Nestin mRNA signature score significantly predicted *BRCA1* germline mutation status, whereas TNP, the basal-like subgroup (by PAM50), CK5, EGFR, and P-cadherin did not (TCGA dataset).

Thus, in addition to being a marker of basal-like features, Nestin might have predictive value in testing breast cancer patients for *BRCA1* germline mutations, although details concerning analytical and clinical validity should be further studied. Also, the practical value of Nestin as an immunohistochemistry based tissue marker might be different between populations.

Nestin expression was associated with high-grade tumour features and reduced survival by multivariate analysis, also when including the triple negative profile. The survival finding was validated in independent cohorts, using mRNA levels and a multigene Nestin expression signature. This is consistent with the observation that a basal-like phenotype is associated with more aggressive behaviour than the hormone receptor positive luminal categories^2^. In line with this, we found that Nestin positivity was associated with increased proliferation, as reported^21, 22^. We also observed that Nestin was significantly associated with activated angiogenesis and blood vessel invasion, as a potential reflection of early haematogenous spread, whereas there was no association with lymphatic involvement. It has been reported by others that basal-like tumours are associated with lymph node negative breast cancer^23^, which was observed in one of our cohorts, and increased presence of brain metastasis^24^.

It was previously suggested that breast cancer may be initiated within a population of stem cells^25^, and that poorly differentiated and highly aggressive tumours display features of cancer stem cells^9^. In normal breast tissue, Nestin is expressed in the myoepithelial or basal layer, which is considered to represent a regenerative compartment^9^, and *BRCA1* has been hypothesized to be a regulator of the normal stem-cell population^26^. When compared with mammary gene signatures reported by Lim *et al*.^17^ Nestin mRNA and signature score significantly correlated with the mammary stem cell enriched and the luminal progenitor signature scores, both of which were enriched in patients with *BRCA1* germline mutations (TCGA dataset). Significant negative correlations were seen with the mature luminal signature score, which was significantly lower in patients with *BRCA1* germline mutations, suggesting that Nestin is present in both the stem and luminal progenitor compartment, but not in mature luminal cells. Furthermore, we found that Nestin was related to KRAS activation and gene expression patterns thought to reflect cancer stemness, such as activation of Wnt/β-Catenin related pathways and retinol metabolism. Recent literature suggests a link between Wnt/β-Catenin and stemness features in cancer^27^, impaired retinol metabolism in cancer stem cells^28^, and a study indicating KRAS as a promoter of mesenchymal properties in basal-like breast cancer^29^. Taken together, these findings support an association between Nestin, stemness and mesenchymal tumour features.

To speculate, Nestin might represent a potential treatment target, especially due to its combined expression on tumour cells and immature tumour vessels^10^. Knockdown of Nestin has shown reduced cell motility in prostate cancer^30^, and also reduced cell proliferation in colorectal^31^, nasopharyngeal^32^, and lung carcinoma^33^.

In conclusion, Nestin was associated with germline *BRCA1* related breast cancer, a basal-like phenotype, as well as high-grade tumour features and reduced survival by multivariate analysis. Also, Nestin was associated with gene expression patterns indicating stem-like tumour features.

## Methods

### Patient series

Nestin protein expression was evaluated in 5 patient series with primary invasive breast carcinoma. Series I and II include women diagnosed with breast cancer (50–69 years at diagnosis) as part of the prospective and population-based Norwegian Breast Cancer Screening Program (NBCSP). Patients in Series I, n = 546, were diagnosed during 1996–2003 (Hordaland County, Norway, ethical approval REK (Regional Committees for Medical and Health Research Ethics, 2014/1984)^34^, and Series II during 2004–2009, n = 282 (Vestfold County, Norway, ethical approval REK 2008/16904)^35^. Series III is a case-control study of 53 patients with *BRCA1* and 45 with *BRCA2* germline mutations collected at McGill University Hospital, Montreal, Canada between 1981 and 2005. 104 *BRCA* non-carriers were included as controls (ethical approval Canada A03-M33-02A and Norway REK 2014/1984) (for details concerning *BRCA* mutation status, see Supplementary Methods). Series IV includes 192 breast cancer patients identified during 1990–2002 in the Kampala Cancer Registry at the Department of Pathology, Makerere University College of Health Sciences (Kampala, Uganda, ethical approval obtained from the Research Ethical Committee at Makerere University College of Health Sciences, and from Norway REK 2014/1984)^36^. Series V includes 35 patients with histologically verified breast cancer distant metastasis during 1996–2007 from Haukeland University Hospital’s archive (Hordaland County, Norway, ethical approval REK 2014/1984). Tissue from primary breast carcinoma and cognate metastasis (liver, bone) was included^37^.

Ethical approval for conducting this research was endorsed by REK, without requiring informed consent. All methods were performed in accordance with guidelines and regulations by the University of Bergen and REK.

Characteristics of the study populations are presented in Supplementary Table S4. Additional information on the patient series and histological variables is presented in Supplementary Methods.

### Nestin protein expression

Immunohistochemical staining of Nestin (10c2 sc-23927, Santa Cruz Biotechnology Inc., Santa Cruz, CA, USA)^5, 9, 38^ was done on 4–5 µm tissue microarray (TMA) sections (Series I-IV), and on standard tissue slides (Series V) of formalin-fixed, paraffin-embedded tissue. Some cases were excluded due to poor quality or insufficient material for evaluation of staining (Series I n = 18, Series II n = 3, Series III n = 21, Series IV n = 5). Nestin protein expression in breast cancer cells was evaluated as either positive or negative. For practical reasons, at least three clearly positive tumour cells were required for a case to be defined as Nestin positive. In addition to breast cancer cells, other cells showed some staining, including endothelial cells, myoepithelial cells of benign breast tissue, nerve tissue, and macrophages. For details, see Supplementary Methods.

### Gene expression and proteomic datasets

Microarray datasets were included for analysis of Nestin mRNA expression and a Nestin gene signature score across breast cancer molecular subtypes (luminal A, B, HER2 enriched, basal-like). Further, Nestin mRNA expression and signature score were analysed in relation to *BRCA1* germline mutations and survival. Upper quartile was used as cut-off because this corresponded best to positive Nestin protein staining.

From The Cancer Genome Atlas (TCGA)^39^ level 3 mRNA microarray data (n = 520) and RNA seq data (n = 1052) from breast cancer samples were downloaded using the TCGA Assembler. TCGA breast cancer proteome data (n = 103) were generated by the Clinical Proteomic Tumour Analysis Consortium (NCI/NIH) using iTRAQ (isobaric Tags for Relative and Absolute Quantification) protein quantification methods (https://cptac-data-portal.georgetown.edu)^39^.

mRNA microarray data generated by the Molecular Taxonomy of Breast Cancer International Consortium (METABRIC) were included (n = 1992)^40^.

From Gene Expression Omnibus (GEO; www.ncbi.nlm.nih/geo), 6 open access breast cancer mRNA microarray datasets with information on molecular subtypes were downloaded: GSE25066 (n = 508)^41, 42^, GSE20685 (n = 327)^43^, GSE40115 (n = 183)^44^, GSE22358 (n = 154)^45^, GSE1456 (n = 159)^46^, GSE25307 (n = 577)^47^. GSE40115 and GSE25307 also provided information on *BRCA1* germline mutation status.

An online database, “Kaplan-Meier plotter” (www.kmplot.com)^48^, was used to evaluate Nestin mRNA expression and signature score in relation to recurrence-free breast cancer survival in a merged dataset (n = 1660).

For additional information of the gene expression datasets and *BRCA* germline mutation status, see Supplementary Methods.

### Gene expression analyses

Differentially expressed genes between cases with Nestin mRNA high versus low expression (cut-off Nestin, upper quartile) in the TCGA dataset were identified based on Significance Analysis of Microarrays (SAM)^49^. Gene sets significantly enriched in Nestin high cases were explored in this cohort, applying the Gene Set Enrichment Analysis (GSEA; www.broadinstitute.org/gsea)^50^ and the signatures of Molecular Signatures Database (MSigDB; www.broadinstitute.org/gsea/msigdb).

Genes differentially expressed between cases of Nestin mRNA high and low (by upper quartile) were incorporated in a 44-gene Nestin signature (39 genes up- and 5 down-regulated in Nestin high cases, TCGA dataset). 2043 genes were found within a false discovery rate of 2% in the SAM-list, and genes from this list with a fold change larger than +/−2.5 were included in the signature (Supplementary Table S5). A Nestin signature score was generated in the mRNA microarray datasets by subtracting the sum of the expression values for the down-regulated genes from the sum of expression values for the up-regulated genes. Also, signature scores (sum of expression values) were generated in the GEO datasets for a selection of the top-ranked signatures significantly enriched in Nestin high cases (TCGA data). Nestin mRNA and signature scores were compared with a mammary stem cell enriched, luminal progenitor, mature luminal, and a stromal signature score, presented by Lim *et al*.^17^ Signature scores were generated by subtracting the sum of the expression values for the down-regulated genes from the sum of expression values for the up-regulated genes. In some of the datasets, a few genes in the signatures (MSigDB/Lim *et al*./Nestin) could not be mapped. 27 of the 44 genes in the Nestin mRNA signature were mapped in the TCGA proteomic dataset, and included in a Nestin protein signature score.

Lehmann *et al*.^16^ published 6 subtypes within the triple-negative group of breast cancer; basal-like 1 and 2, immunomodulatory, mesenchymal, mesenchymal stem-like, and luminal androgen receptor. To assess whether Nestin mRNA levels and signature scores were associated with any of these subtypes, we uploaded gene expression values for the basal-like subtype from two of the largest gene expression cohorts in this study (TCGA n = 89, and GSE25066 n = 167) to the TNBC type online predictor (http://cbc.mc.vanderbilt.edu/tnbc)^51^.

The Topology Enrichment Analysis frameworK (TEAK) subpathway enrichment tool^52^ was applied on 263 samples with the highest Nestin mRNA expression (upper quartile) and 263 samples with the lowest (lower quartile) from the TCGA RNA seq data (n = 1052). The “case-control” setting (case-high, control-low) was implemented and subpathway topologies from metabolic and non-metabolic KEGG pathways were examined.

For details concerning gene expression analyses, see Supplementary Methods.

### Statistical analysis

Data were analysed using SPSS (version 22.0, IBM corp., Armonk, NY, USA). Associations between categorical variables were evaluated by Pearson’s chi-square (χ^2^) test or Fisher’s exact test. Odds ratios (OR) and 95% confidence intervals (CI) are presented. For paired data (primary tumours and corresponding metastasis, Series V), McNemar’s test was used. Spearman’s rank correlation test was applied when comparing bivariate continuous variables, and Spearman’s correlation coefficients (ρ) are reported. When analysing differences in age distributions in the patient series (Series I-IV), Mann-Whitney U test was applied.

Multiple logistic regression, enter method and p-values by the Wald test, was applied for prediction of *BRCA1* germline mutation status and the basal-like phenotype. For *BRCA1* analyses, patients with *BRCA2* mutations were excluded.

Differences in Nestin mRNA expression, protein expression, mRNA signature score and protein signature score across molecular subgroups, and Nestin mRNA and signature score across the triple-negative categories by Lehmann *et al*.^16^, were tested by the Mann-Whitney U and Kruskal-Wallis tests and presented by error-bars with 95% CI of the mean.

For univariate survival analysis, with recurrence and death from breast cancer as end-points, the Kaplan-Meier product-limit method (log-rank test) were applied. Multivariate breast cancer specific survival analysis was performed by Cox’ proportional hazards regression model, with calculations done according to the enter method. Basic prognostic characteristics (tumour diameter, histological grade, lymph node status), TNP, and Nestin were included after evaluating their log-minus-log plot. Significant interactions between the variables were tested by adding interaction terms (a × b) in the regression model. For multivariate analyses, only patients with information on all variables were included.

All statistical tests were two-sided, and statistical significance was assessed at 5% level, and p-values between 5–10% were regarded as borderline significant.

## Electronic supplementary material

Supplementary Information
