# Supplementary material for: Expression of Nestin associates with BRCA1 mutations, a basal-like phenotype and aggressive breast cancer
Source: Sci Rep. 2017 Apr 24;7:1089. doi: 10.1038/s41598-017-00862-w (PMC5430803; doi:10.1038/s41598-017-00862-w)
Supplement: Supplementary file 1 — Supplementary Information [file 41598_2017_862_MOESM1_ESM.pdf]

Supplementary Information

Expression of Nestin associates with *BRCA1* mutations, a basal-like phenotype and aggressive breast cancer

Kristi Krüger[1], Elisabeth Wik[1,2], Gøril Knutsvik[1,2], Hawa Nalwoga[1,3], Tor Audun Klingen[1,4], Jarle B. Arnes[1,2], Ying Chen[1,4,5], Monica Mannelqvist[1], Konstantina Dimitrakopoulou[6], Ingunn M. Stefansson[1,2], Even Birkeland[1], Turid Aas[8], Nicholas P. Tobin[7], Inge Jonassen[6], Jonas Bergh[7], William D. Foulkes[9], Lars A. Akslen[1,2].

[1] Centre for Cancer Biomarkers CCBIO, Department of Clinical Medicine, Section for Pathology, University of Bergen, Bergen, Norway

[2] Department of Pathology, Haukeland University Hospital, Bergen, Norway

[3] Department of Pathology, Makerere University College of Health Sciences, P. O. Box 7072, Kampala, Uganda

[4] Department of Pathology, Vestfold Hospital, Tønsberg, Norway

[5] Department of Pathology, Akershus University Hospital, Lørenskog, Norway

[6] Centre for Cancer Biomarkers CCBIO and Computational Biology Unit, Department of Informatics, University of Bergen, Norway

[7] Department of Oncology and Pathology, Karolinska Institute and University Hospital, Stockholm, Sweden

[8] Department of Surgery, Haukeland University Hospital, Bergen, Norway

[9] Program in Cancer Genetics, Departments of Oncology and Human Genetics, McGill University, 546 Pine Avenue West, Montreal, QC H2W 1S6, Canada

Supplementary Methods

PATIENTS

Patient series

Characteristics of the study populations are presented in Supplementary Table S4 online. Series I and II were similar in regard to age at diagnosis, histological type, lymph node status, and receptor status. Compared to Series I, cases in Series III and IV had more aggressive tumours with features such as higher histological grade, higher proportion of hormone receptor negativity and lower age at diagnosis.

Series I

This series includes 546 women (50–69 years at time of diagnosis) diagnosed with primary invasive breast cancer (403 screen-detected, 143 interval-detected cancers) as part of the prospective and population-based Norwegian Breast Cancer Screening Program (NBCSP) during 1996-2003 (Hordaland County, Norway; 10% of the Norwegian population). Patients with distant metastatic disease at time of diagnosis (stage IV) were not included. The patients received treatment according to well standardized national protocols in a single institution. Follow-up information was given by the Norwegian Cause of Death Registry, and can be considered accurate and complete. Last date of follow-up was December 31, 2011. Outcome data includes survival status, survival time and cause of death. During the follow-up period, 79 patients (15%) died from breast carcinoma, and 62 (12%) died from other causes. The 5-year breast cancer specific mortality was 7% (37/546). The median follow-up time for survivors was 13 years. Histological slides were

reviewed by G.K.[1]. The study was approved by the Western Regional Committee for Medical and Health Research Ethics, REC West (REK 2014/1984).

Series II

This series includes 282 women (50–69 years at time of diagnosis) diagnosed with primary invasive breast cancer (199 screen-detected, 83 interval-detected cancers) as part of the NBCSP during 2004-2009 (Vestfold County, Norway; 5% of the Norwegian population). Follow-up information was collected from medical records, and the last follow-up was in June, 2013. During the follow-up period, 24 patients (8%) died from breast carcinoma, and 8 (3%) died from other causes. 5-year breast cancer specific mortality was 5% (14/282). The median follow-up time for survivors was 6 years. Histological slides were reviewed by T.A.K.[2]. The study was approved by the South-Eastern Regional Committee for Medical and Health Research Ethics, REC South-East (REK 2008/16904).

Series III

This series is a case-control study consisting of 53 patients with *BRCA1* and 45 with *BRCA2* germline mutations, and 53 *BRCA* non-carriers collected at McGill University Hospital, Montreal, Canada. Breast cancer samples were collected from affected members of families counselled at the Hereditary Cancer Clinics of McGill University between 1981 and 2005. All cases were referred because of a personal or family history of breast or ovarian cancer and were offered *BRCA1/2* testing. The study was approved by the

Institutional Review Board, McGill University Hospital, A03-M33-02A. In addition, 51 randomly selected control cases from a series of *BRCA* non-carriers were included (information on *BRCA* status was obtained from Dr. Apold, Department of Genetics, Haukeland University Hospital, Bergen, Norway). The three groups were balanced with respect to age and tumour size, but a strict case-by-case matching procedure could not be accomplished. Slides were reviewed by J.B.A. The study was approved by the Western Regional Committee for Medical and Health Research Ethics, REC West (REK 2014/1984).

Series IV

Cases of primary breast carcinoma with available and technically suitable archival paraffin blocks from the period 1990-2002 were identified in the Kampala Cancer Registry at the Department of Pathology, Makerere University College of Health Sciences, Kampala, Uganda. Altogether, 192 cases were included in the study. The registry methods of collecting data and results have been reported previously[3]. Clinical information was obtained from histology reports, and slides were reviewed by H.N.[4]. The permission to conduct this research was obtained from the Research Ethical Committee at Makerere University College of Health Sciences and from the Western Regional Committee for Medical and Health Research Ethics, REC West, Norway (REK 2014/1984).

Series V

This series includes 35 patients with histologically verified breast cancer metastasis during 1996-2007 from Haukeland University Hospital's archive, Hordaland County, Norway. Tissue from the primary breast carcinoma and cognate metastasis (liver, bone) was collected. Slides were reviewed by I.M.S.[5]. The study was approved by the Western Regional Committee for Medical and Health Research Ethics, REC West (REK 2014/1984).

Gene expression datasets

The Cancer Genome Atlas datasets

Using The Cancer Genome Atlas (TCGA) Assembler[6], level 3 mRNA microarray data (n=520) and RNA seq data (n=1052) from invasive breast cancer samples were downloaded. The RNA seq data was mapped according to the HGNC gene symbol nomenclature, and RNA seq RSEM normalized data were analysed. Molecular subtypes were determined by the PAM50 algorithm[7], as published in the primary TCGA breast cancer publication[8].

Molecular Taxonomy of Breast Cancer International Consortium dataset

This study uses data generated by The Molecular Taxonomy of Breast Cancer International Consortium (METABRIC). Funding for the project was provided by Cancer Research UK and the British Columbia Cancer Agency Branch[9]. The METABRIC microarray dataset includes 1992 breast cancer samples, and molecular subtypes were defined by the PAM50 classification[7].

Gene Expression Omnibus datasets

From Gene Expression Omnibus (GEO; www.ncbi.nlm.nih/geo), we downloaded 6 open access normalized and log-transformed breast cancer microarray datasets, with information on molecular subtypes. The following cohorts were used: GSE25066 (n=508)[10,11], GSE20685 (n=327)[12], GSE40115 (n=183)[13], GSE22358 (n=154)[14], GSE1456 (n=159)[15], and GSE25307 (n=577)[16]. GSE40115 and GSE25307 also provided information on *BRCA* germline mutation status.

GSE25066

This dataset includes 508 patients with HER2 (human epidermal growth factor receptor 2) negative breast cancer (stage I-III). Intrinsic molecular subtypes, based on PAM50 classification[7], are available[10,11].

GSE20685

This dataset includes 327 breast cancer patients (stage I-IV). Molecular subtypes were divided into six, I-VI. Type I is similar to the basal-like subtype; II is similar to the HER2 enriched subtype; III represents a mixture of HER2 and luminal B subtypes; IV represents luminal B, and V-VI represent the luminal A subtype[12,17,18].

GSE40115

This dataset includes 183 patients, 33 with *BRCA1* germline mutations, 22 with *BRCA2* germline mutations, and 128 randomly chosen sporadic tumours[13]. In addition to *BRCA1/2* germline status, molecular subtypes based

on PAM50[7] classification are available.

GSE22358

This dataset includes 154 breast cancer patients (stage II-III)[14], with molecular subtypes based on PAM50 classification[7].

GSE1456

This dataset is from a series of 159 breast cancer patients (stage I-II)[15]. Molecular subtypes were classified as described by Sørlie et al.[17,18]. The gene expression profiling of this cohort has previously been approved by the Ethics Committee at the Karolinska University Hospital together with additional amendments.

GSE25307

This dataset includes 577 patients, 34 with *BRCA1* and 39 with *BRCA2* germline mutations, 195 with familial non-*BRCA*-mutated cancer, and 273 sporadic tumours[16]. Molecular subtypes were based on PAM50 classification[7]. A probe presence filter had been applied to select only probes present in at least 520 of 577 assays (n=10377)[16]. Thus, signature generation was not feasible, since many of the relevant probes were not present. Only Nestin mRNA expression and its association with molecular subtypes and *BRCA1* germline mutations were analysed in this dataset.

Kaplan-Meier online database

An online database, “KM-plotter” (www.kmplot.com)[19], including EGA

(European Genome-phenome Archive) and GEO datasets (Affymetrix microarrays only), was used to evaluate Nestin mRNA levels and signature score in relation to recurrence-free breast cancer survival in a merged dataset of 1660 breast cancer cases.

BRCA germline mutation status

For *BRCA1/2* germline mutation status of Series III, DNA was extracted from paraffin-embedded tissue, and mutation analysis was carried out as previously described, looking specifically for the recurrent mutations in the Ashkenazi Jewish population (*BRCA1*: c.68_69delAG (185delAG), c.5266dupC (5382insC); *BRCA2*: c.5946delT (6174delT))[20,21]. In the Norwegian population, four known founder mutations were tested for (*BRCA1*: c.1556delA (1675delA), c.1011dupA (1135insA), c.697_698delGT (816delGT), c.3228_3229delAG (3347delAG)).

BRCA1/2 germline mutation status in DNA from normal tissue in the TCGA dataset on invasive breast cancer was accessed through the TCGA data portal (controlled access data)[8], and is in accordance with *BRCA1* germline mutation carriers presented by Prat et al.[22]. Current status for clinical significance was checked in the ClinVar database (http://www.ncbi.nlm.nih.gov/clinvar) for all *BRCA1* germline mutations. 2 patients previously considered as being *BRCA1* mutation carriers had a mutation (c.2521C>T) that is regarded as benign, and were re-classified to *BRCA* non-carriers, leaving 11 *BRCA1* germline mutated patients for analysis. *BRCA1* mutations included: c.181T>G, c.2800C>T, c.68_69delAG, c.5095C>T, c.329dupA, c.594-2A>C, c.5137delG, c.5266dupC, c.4035delA,

c.3967C>T. For validation of *BRCA1* findings, the datasets GSE40115[13] and GSE25307[16] were included.

METHODS

Immunohistochemistry

Staining was done on 4-5 µm tissue microarray (TMA) sections of formalin-fixed and paraffin-embedded tissue in Series I-IV, on standard tissue slides in Series V and cases from Series I-II with poor quality or insufficient material for evaluation of staining in the TMAs.

Pre-treatment of the sections was done, *i.e.* pressure cooker antigen retrieval in Target Retrieval Solution pH 6.0 (Dako S1699, Glostrup, Denmark), and 8 min incubation with Peroxidase Blocking Reagent (Dako S2001). The sections were incubated for 60 min at room temperature with monoclonal mouse Nestin antibody (10c2 sc-23927, Santa Cruz Biotechnology Inc., Santa Cruz, CA, USA)[23-25] diluted 1:50 in Series III-IV, and 1:200 in Series I-II, V, and on the standard tissue sections, in antibody diluent (Dako K8006). Horseradish peroxidase EnVision rabbit/mouse (Dako K5007) was applied for 30 min at room temperature. Nestin was visualized by applying Liquid Dab+ Substrate Chromogen System (Dako K4011) for 10 min at room temperature. Counterstaining was done using Haematoxylin (Dako S2020) for 3 min. Positive and negative controls were included in each staining round. Basal-like breast cancer cases, also including benign epithelial tissue, were used as controls. In addition to Nestin expression in cancer cells, Nestin staining was seen in myoepithelial and endothelial cells, as expected.

The same breast cancer cases were used as negative controls, adding diluent instead of the Nestin antibody.

Inter- and intra-observer agreement for Nestin immunostaining

For estimation of inter-observer agreement regarding Nestin staining, Series IV was scored separately by an experienced pathologist (H.N.), and Kappa (κ) statistics was calculated; κ=0.88. Intra-observer (K.K.) agreement was calculated for all 5 series, Series I; κ=0.95, Series II; κ=0.82, Series III; κ=0.92, Series IV; κ=0.86, Series V; primary tumour κ=1.0, and metastasis κ=0.85.

Western Blot

For evaluation of antibody specificity, Western Blot analysis of Nestin (10c2 sc-23927, Santa Cruz Biotechnology Inc., Santa Cruz, CA, USA)[23-25], dilution 1:1000 and incubation overnight at 4°C, was done on whole cell lysates from ten breast cancer cell lines (6 basal, 4 luminal; MB-468, BT-549, MD-231, SUM 1315, SUM 159, HS 578T, MCF7, ZR-75-30, BT-474, T47D). A distinct band was visualized at expected size, about 220 kDa in all cell lines (not shown).

Variables and cut-off values

Data on several variables were available from previous studies, Series I[1,26-28], Series II[2,29], Series IV[4,30]. For Series III, markers were determined as previously published[20,28,31-35].

In general, cut-off values for continuous variables were determined based on median or quartile values, also considering the distribution profile, the size

of subgroups, and number of events in survival analyses. Immunohistochemical (IHC) staining of ≥10% of the tumour nuclei was regarded as a positive oestrogen and progesterone receptor (ER and PR) status in these studies, according to national guidelines at the time of diagnosis[26,29,36,37]. HER2 immunostaining was scored according to the Dako Herceptest criteria[38], considering the intensity and degree of membranous reaction. In all series, a HER2 IHC score of 3+ was considered positive, in Series III and IV, a score less than 3+ was considered negative[34,36,38]. In Series I and II, a membranous reaction of 2+ was evaluated by SISH or CISH, respectively, and the cases were regarded positive if the HER2/Chr17 ratio by SISH/CISH was ≥ 2.0[1,29].

Ki-67 staining (nuclear reactivity) was assessed according to the approach of Weidner et al.[39], counting the proportion of positive tumour cell nuclei in the most active areas (hot-spots). In this study, the series specific upper quartile was used as cut-off values for high proliferation (31.5, 23.0, and 38.7% in Series I, II, and IV, respectively)[1,4,29,36]. Ki-67 values were not available in Series III, and mitosis count, divided by the upper quartile (18.5 in 10 high power fields, 11.6 mitoses/mm^2) was used.

EGFR (epidermal growth factor receptor) was evaluated according to the Dako EGFR PharmDX kit instructions (Dako Corp., Carpinteria, CA, USA), where membranous reactivity above background in >1% of tumour cells was considered positive[28,40].

The degree of positivity for several of the variables was estimated by Staining Index (SI) values 0-9[41]. For CK5 (Cytokeratin 5), SI 1-9 was regarded

as positive, whereas for p53 and P-cadherin, a positive staining was defined as SI 4-9[4,26,32,34,36].

Based on immunohistochemistry, the core basal phenotype (CBP; ER- HER2- CK5+ and/or EGFR+)[42], and five basal-like profiles were defined, BLP 1-5, as previously described[4,28].

For estimation of activated angiogenesis, the proportion of vessels with proliferating endothelial cells, vascular proliferation index (VPI) was used, with series specific upper quartile as cut-off values (5.44, 2.25 and 3.10%, Series I, III, IV, respectively)[30,33,43,44]. Blood or lymph vessel invasion (BVI, LVI)[2], and glomeruloid microvascular proliferation (GMP)[27,31], were scored within the tumour as either present or absent.

Gene expression microarray analyses

Differentially expressed genes between cases with Nestin mRNA high versus low expression (cut-off Nestin, upper quartile) in the TCGA dataset were identified based on Significance Analysis of Microarrays (SAM)[45]. Gene sets significantly enriched in Nestin high cases were explored in this cohort, applying the Gene Set Enrichment Analysis (GSEA; www.broadinstitute.org/gsea)[46], and the signatures of Molecular Signatures Database (MSigDB; www.broadinstitute.org/gsea/msigdb). The gene expression datasets were imported to J-Express/2012 (www.molmine.com)[47,48] for performance of SAM and GSEA. The max probe option in J-Express was selected in cases of multiple probes per gene symbol.

GSEA/MSigDB results were sorted by false discovery rates (FDR) from lowest to highest. In the "Hallmark signatures" list, [HALLMARK_WNT_BETA_CATENIN_SIGNALING] was top-ranked and selected for further analysis. 21 KRAS signatures were found within FDR 25% in the C6 list, "Oncogenic signatures", 17 of them within FDR 10%. The signature [KRAS.300_UP.V1_UP] was top-ranked and selected for further analysis.

Nestin mRNA and signature scores were compared with a mammary stem cell enriched, luminal progenitor, mature luminal, and a stromal signature score, presented by Lim et al.[49]. Signature scores were generated by subtracting the sum of the expression values for the down-regulated genes from the sum of expression values for the up-regulated genes.

RNA seq analysis-TEAK

With the use of TCGA Assembler[6], the RNA seq level 3 RSEM normalized data from 1052 tumour samples were downloaded and processed. From this cohort, 263 samples with the highest expression of Nestin mRNA (upper quartile) and 263 samples with the lowest expression (lower quartile) were retained for subpathway enrichment analysis. We filtered genes with zero counts in more than 80% of the samples. An offset of 1 was added to normalized counts and log2 transformation was applied.

The Topology Enrichment Analysis frameworK (TEAK) tool[50] was applied after downloading the updated human KEGG pathway KGML files[51]. The tool, with default parameters, was run and the "case – control" setting was implemented. "Cases" were defined as the samples with high Nestin

expression and as “controls”, the samples with low Nestin expression. All linear and non-linear subpathway topologies from both metabolic and non-metabolic KEGG pathways were examined.

Triple-negative subtypes

To assess whether Nestin mRNA levels and signature score were associated with the triple-negative categories published by Lehmann et al.[52], we uploaded gene expression values for the basal-like subtype (by PAM50 classification[7]) from two of the largest gene expression cohorts in this study (TCGA and GSE25066) to the TNBC type online predictor (http://cbc.mc.vanderbilt.edu/tnbc)[53]. Because of positive ER status, 6 and 22 cases from the basal-like group from TCGA and GSE25066 could not be included in the TNBC predictor tool, leaving 89 cases from TCGA and 167 cases from GSE25066 for analysis.

TCGA Proteomic analysis

TCGA breast cancer proteome dataset (n=105), log ratio expression values, was imported to J-Express/2012 (www.molmine.com)[47,48], and proteins with missing values in more than 50% of the samples were excluded. The remaining missing values were then imputed using the LSImpute Adaptive option[54]. 27 of the 44 genes in the Nestin mRNA signature were mapped in the TCGA proteomic dataset, and included in a Nestin protein signature score. If excluding cases with missing protein values in 30% of the samples, only 22 of 44 proteins were mapped. However, this protein signature was also strongly correlated to the 44-gene signature (Spearman’s $\rho=0.81$, $p<0.0005$).

The proteome data was merged with the mRNA microarray data (n=520), giving 103 patients with information on both protein and mRNA expression, as well as clinico-pathological data.

Statistical analysis

Data were analysed using the SPSS (version 22.0, IBM corp., Armonk, NY, USA). Associations between categorical variables were evaluated by Pearson's chi-square (χ^2) test or Fisher's exact test, and odds ratios (OR) and 95% confidence intervals (CI) are presented. For paired data (primary tumours and corresponding metastasis, Series V) McNemar's test was used. Kappa coefficients (κ) are presented for inter- and intra-observer agreement regarding Nestin immunostaining. Spearman's rank correlation test was applied when comparing bivariate continuous variables, and Spearman's correlation coefficients (ρ) are reported. When analysing differences in age distributions in the patient series (Series I-IV), Mann-Whitney U test was applied.

Concerning prediction of *BRCA1* germline mutation status, Nestin protein expression or mRNA expression or Nestin mRNA signature score was examined in addition to basal-like associated markers CK5, EGFR, and P-cadherin, the triple negative profile (TNP), and the basal-like phenotype (by PAM50), by multiple logistic regression, using the enter method and p-values calculated by the Wald test. Only patients with information on all variables were included in the analysis. For *BRCA1* analyses, patients with *BRCA2* mutations were excluded. Multiple logistic regression, enter method and Wald test for significance, was also used for prediction of the basal-like subtype.

Nestin mRNA expression/protein expression/mRNA signature score/protein signature score was included in addition to CK5, EGFR, and P-cadherin. Only patients with information on all variables were included in the analysis.

Differences in Nestin mRNA, protein expression, mRNA signature score, and protein signature score across molecular subgroups, and Nestin mRNA and mRNA signature score across the 6 triple-negative categories by Lehmann et al.[52], were tested by the Kruskal-Wallis test or Mann-Whitney U test and presented by error-bars with 95% confidence interval of the mean.

For univariate survival analysis, with death from breast cancer as end-point, the Kaplan-Meier product-limit method (log-rank test) were applied. Multivariate breast cancer specific survival analysis was performed by Cox' proportional hazards regression model, with calculations done according to the enter method. Basic prognostic characteristics (tumour diameter, histological grade, lymph node status), the TNP, and Nestin, were included after evaluating their log-minus-log plot. Significant interactions between the variables were tested by adding interaction terms (a x b) in the Cox' regression model. Only patients with information on all variables were included in the analysis.

The association between recurrence-free breast cancer survival and Nestin mRNA expression and signature score in a merged breast cancer dataset was performed using the Kaplan-Meier Plotter online survival tool (kmplot.com)[19], and separate Kaplan-Meier plots were made for Nestin mRNA expression and Nestin signature score (upper quartile as cut-off values). For the Nestin signature, 41 of the 44 signature genes were found in the kmplot database, and included in the multigene classifier (mean probe expression),

and JetSet filter[55] for probe set quality was applied. In the METABRIC cohort, breast cancer specific survival according to Nestin mRNA expression and Nestin signature score (42 of 44 genes were found) was evaluated by Kaplan-Meier product-limit method and p-values by the log-rank test are presented (upper quartile as cut-off).

All statistical tests were two-sided, and statistical significance was assessed at 5% level, and p-values between 5-10% were regarded as borderline significant.

Legends to supplementary figures

Supplementary Figure S1A. Microarray data of Nestin mRNA (*NES*) expression across breast cancer molecular subtypes.

Nestin mRNA expression (*NES*) is presented by error-bars with 95% confidence interval of the mean, with p-values by the Kruskal-Wallis test. The normal breast-like category was excluded. METABRIC (n, number of patients=1784), GSE25066 (n=464), GSE20685 (n=327), GSE40115 (n=180), GSE22358 (n=139), GSE1456 (n=102), GSE25307 (n=447).

Supplementary Figure S1B. Microarray data of Nestin mRNA signature score across breast cancer molecular subtypes.

The Nestin signature score is presented by error-bars with 95% confidence interval of the mean, with p-values by the Kruskal-Wallis test. The normal breast-like category was excluded. METABRIC (n, number of patients=1784), GSE25066 (n=464), GSE20685 (n=327), GSE40115 (n=180), GSE22358 (n=139), GSE1456 (n=102).

Supplementary Figure S2. Univariate breast cancer specific survival according to Nestin status in TNP strata and multivariate survival analysis.

Kaplan-Meier univariate breast cancer specific survival analysis in Series I according to Nestin protein expression (log-rank test for difference) in triple negative profile (TNP) absent strata (A) and TNP present strata (B). For each category, the number of breast cancer deaths is given, followed by the total number of cases in each category.

Supplementary Figure S3. Recurrence-free breast cancer survival by Nestin mRNA expression and Nestin signature score.

Kaplan-Meier recurrence-free breast cancer survival according to Nestin mRNA expression (left) and Nestin signature score (right) from www.kmplot.com[19], with upper quartile as cut-off value.

Supplementary Figure S4A-D. Nestin mRNA expression and Nestin mRNA signature score compared with Wnt/β-catenin and KRAS signature scores.

Microarray data of Nestin mRNA (*NES*) expression (A) and Nestin signature score (B) compared with Wnt/β-catenin signature score, and Nestin mRNA expression (C) and Nestin signature score (D) compared with KRAS signature score. The normal breast-like category was excluded. P-values by Spearman's rank correlation and the coefficients (ρ) are presented. TCGA (n, number of patients=505), GSE25066 (n=464), GSE20685 (n=327), GSE40115 (n=180), GSE22358 (n=139), GSE1456 (n=102). Molecular subtypes are indicated with colours; basal-like: blue; HER2 enriched: green; luminal A: orange; luminal B; red. In GSE20685, molecular subtypes I-IV is indicated as follows; I: blue; II: green; III: turquoise; IV: red; V: orange; VI: light green/yellow.

Supplementary Figure S5. Microarray data of Nestin mRNA (*NES*) expression and Nestin signature score across triple-negative breast cancer subtypes.

Microarray data of Nestin mRNA (*NES*) expression and Nestin signature score across triple-negative subtypes according to Lehmann et al.[52], presented by error-bars with 95% confidence interval of the mean, and p-values by the Kruskal-Wallis test. BL1: basal-like 1; BL2: basal-like 2; IM: immunomodulatory; M: mesenchymal; MSL: mesenchymal stem-like; LAR: luminal androgen receptor. TCGA (n, number of patients=89), GSE25066 (n=167).

Supplementary Figure S6. Nestin mRNA expression and mRNA signature score, compared with mammary stem cell enriched, luminal progenitor, mature luminal, and stromal signature scores.

TCGA microarray data (n=505) of Nestin mRNA (*NES*) expression and Nestin signature score compared with mammary stem cell enriched, luminal progenitor, mature luminal, and stromal signature scores, according to Lim et al.[49]. The normal breast-like category was excluded. P-values by Spearman's rank correlation and the coefficients (ρ) are presented. Molecular subtypes are indicated with colours; basal-like: blue; HER2 enriched: green; luminal A: orange; luminal B; red. Abbreviations: MaSC: mammary stem cell; Sign: signature; Lum: luminal; Prog: progenitor.

Figure S1A

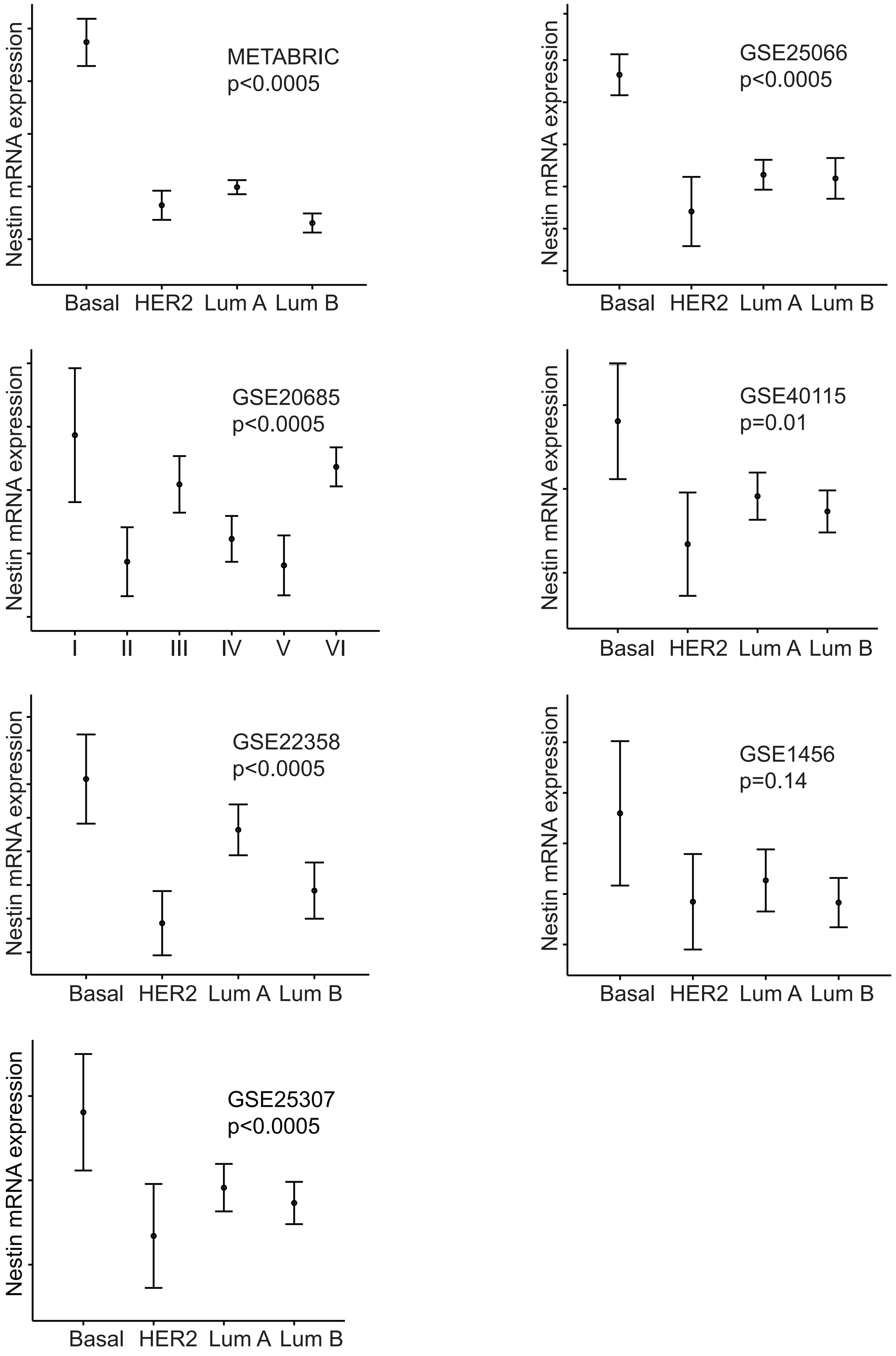
Nestin mRNA expression
METABRIC
p<0.0005
Basal
HER2
Lum A
Lum B
GSE25066
p<0.0005
GSE20685
p<0.0005
I
II
III
IV
V
VI
GSE40115
p=0.01
GSE22358
p<0.0005
GSE1456
p=0.14
GSE25307
p<0.0005

Figure S1B

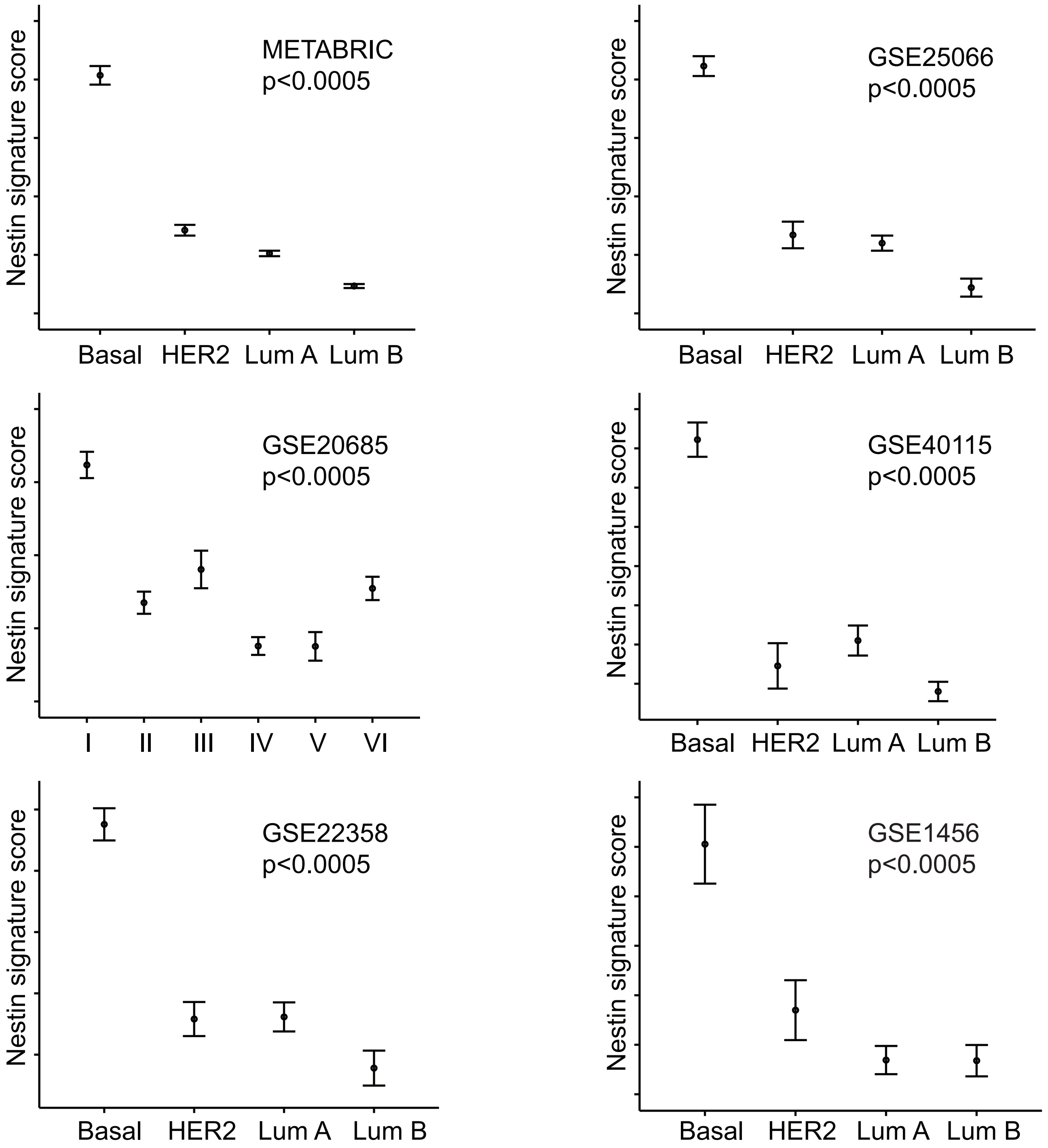
METABRIC
p<0.0005
Nestin signature score
Basal
HER2
Lum A
Lum B
GSE25066
p<0.0005
Nestin signature score
Basal
HER2
Lum A
Lum B
GSE20685
p<0.0005
Nestin signature score
I
II
III
IV
V
VI
GSE40115
p<0.0005
Nestin signature score
Basal
HER2
Lum A
Lum B
GSE22358
p<0.0005
Nestin signature score
Basal
HER2
Lum A
Lum B
GSE1456
p<0.0005
Nestin signature score
Basal
HER2
Lum A
Lum B

Figure S2

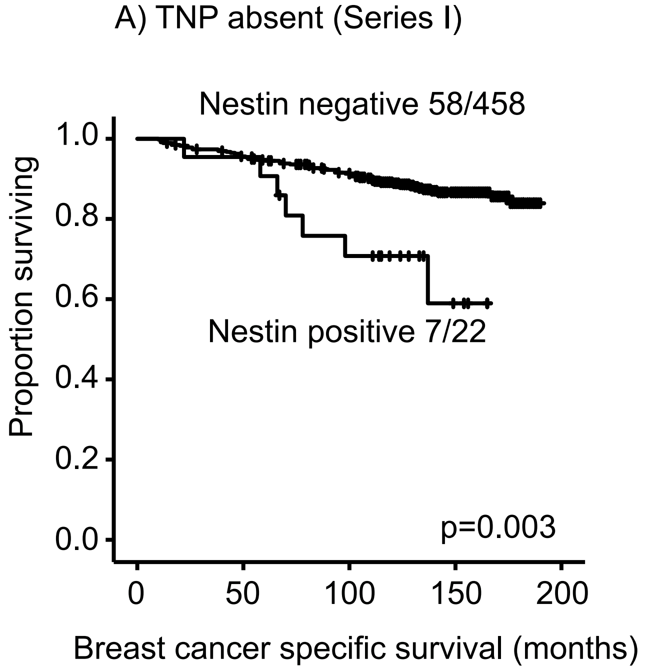

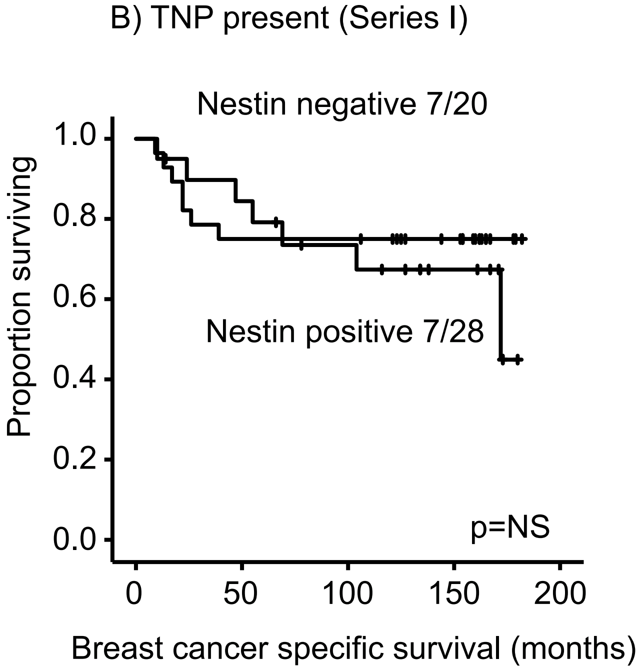

Univariate and multivariate survival analysis (Cox' proportional hazards regression) with death from breast cancer as end-point (Series I).

Variables	n	Univariate HR (95% CI)	*P*	Multivariate HR (95% CI)	*P*
Tumour diameter					
< 2.0 cm	358	1.0		1.0	
≥ 2.0 cm	165	3.7 (2.3-5.9)	<0.0005	2.3 (1.4-3.8)	0.001
Histological grade					
1-2	433	1.0		1.0	
3	90	2.4 (1.5-3.9)	<0.0005	1.3 (0.7-2.3)	NS
Nodal status					
Negative	382	1.0		1.0	
Positive	141	4.7 (3.0-7.4)	<0.0005	3.7 (2.2-6.0)	<0.0005
Nestin					
Negative	473	1.0		1.0	
Positive	50	2.5 (1.4-4.5)	0.002	2.4 (1.1-5.5)	0.034
TNP					
Absent	475	1.0		1.0	
Present	48	2.6 (1.5-4.7)	0.001	3.3 (1.5-7.5)	0.004
				1.0	
Nestin * TNP				0.3 (0.1-1.0)	0.055

n: number of patients; HR: hazard ratio; CI: confidence interval; P: p-values; NS: not significant; TNP: triple negative profile. Only patients with information on all variables were included in the analysis (n=523).

Figure S3

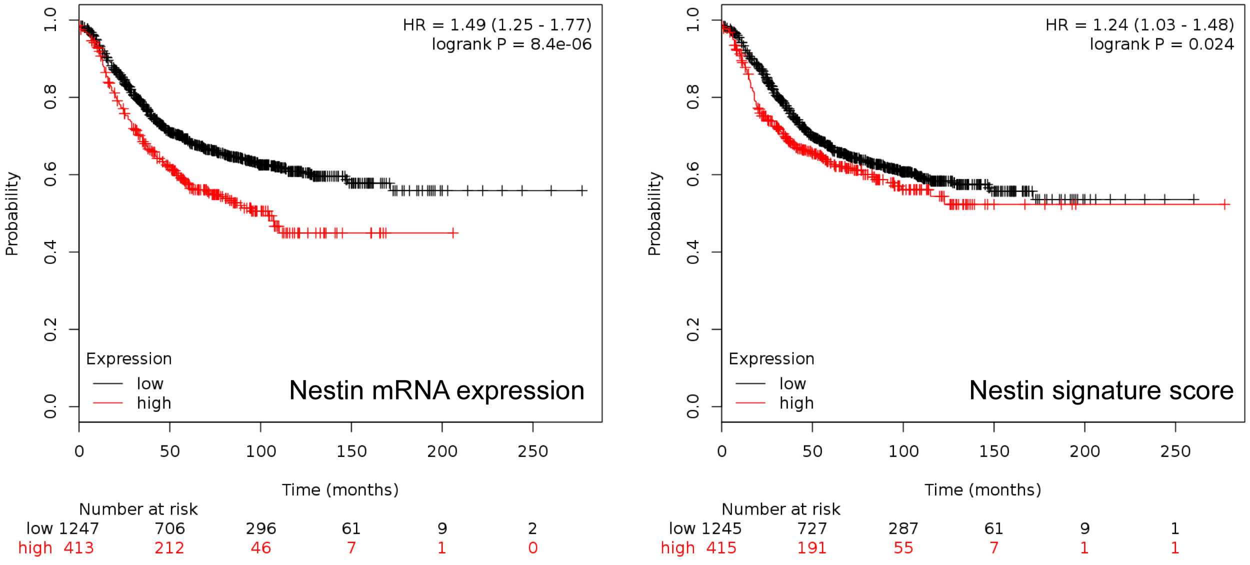
HR = 1.49 (1.25 - 1.77)
logrank P = 8.4e-06
Probability
Expression
low
high
Nestin mRNA expression
Time (months)
Number at risk
low 1247 706 296 61 9 2
high 413 212 46 7 1 0
HR = 1.24 (1.03 - 1.48)
logrank P = 0.024
Probability
Expression
low
high
Nestin signature score
Time (months)
Number at risk
low 1245 727 287 61 9 1
high 415 191 55 7 1 1

Figure S4A

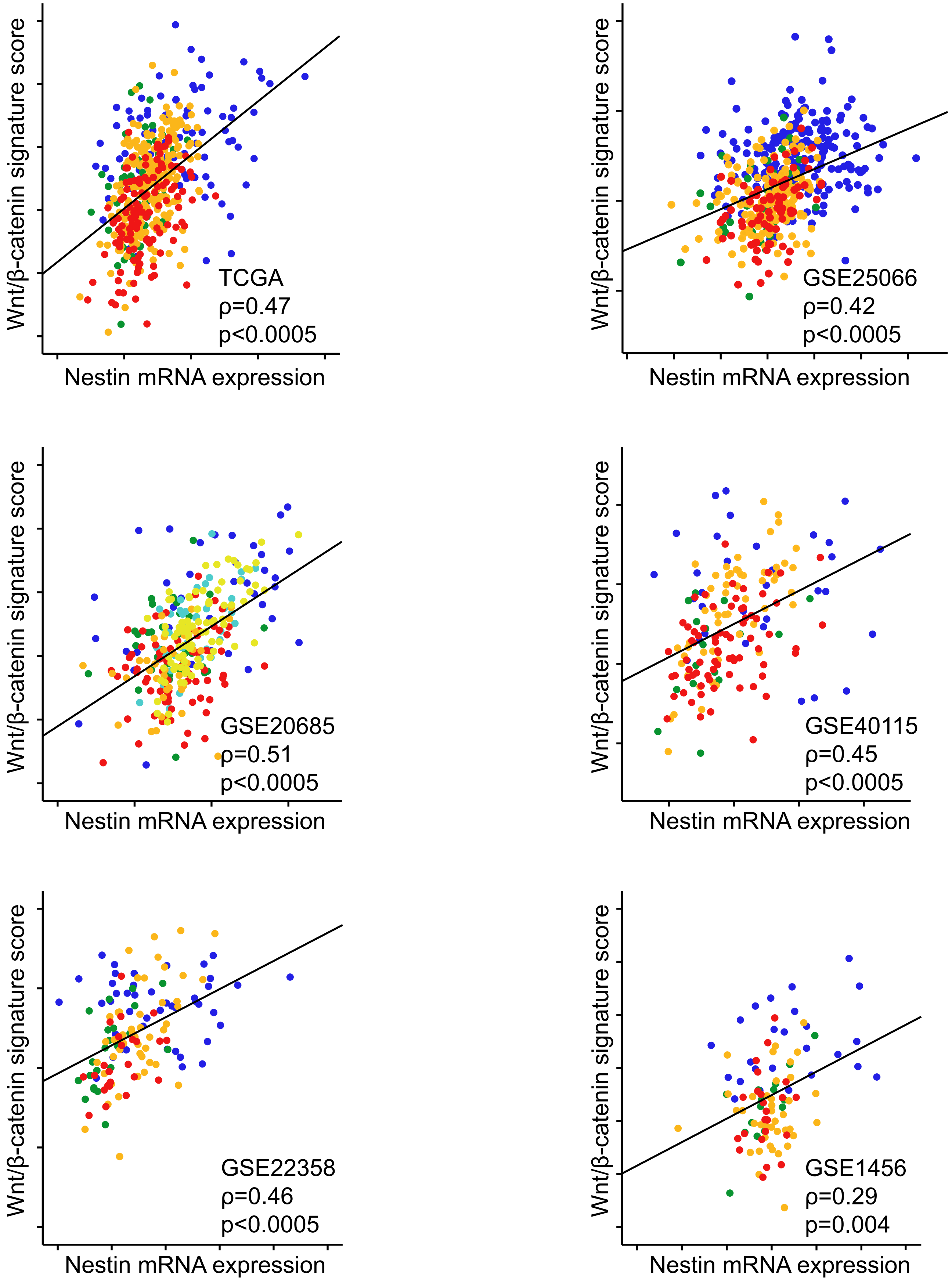
Wnt/β-catenin signature score
Nestin mRNA expression
TCGA
ρ=0.47
p<0.0005
GSE25066
ρ=0.42
p<0.0005
GSE20685
ρ=0.51
p<0.0005
GSE40115
ρ=0.45
p<0.0005
GSE22358
ρ=0.46
p<0.0005
GSE1456
ρ=0.29
p=0.004

Figure S4B

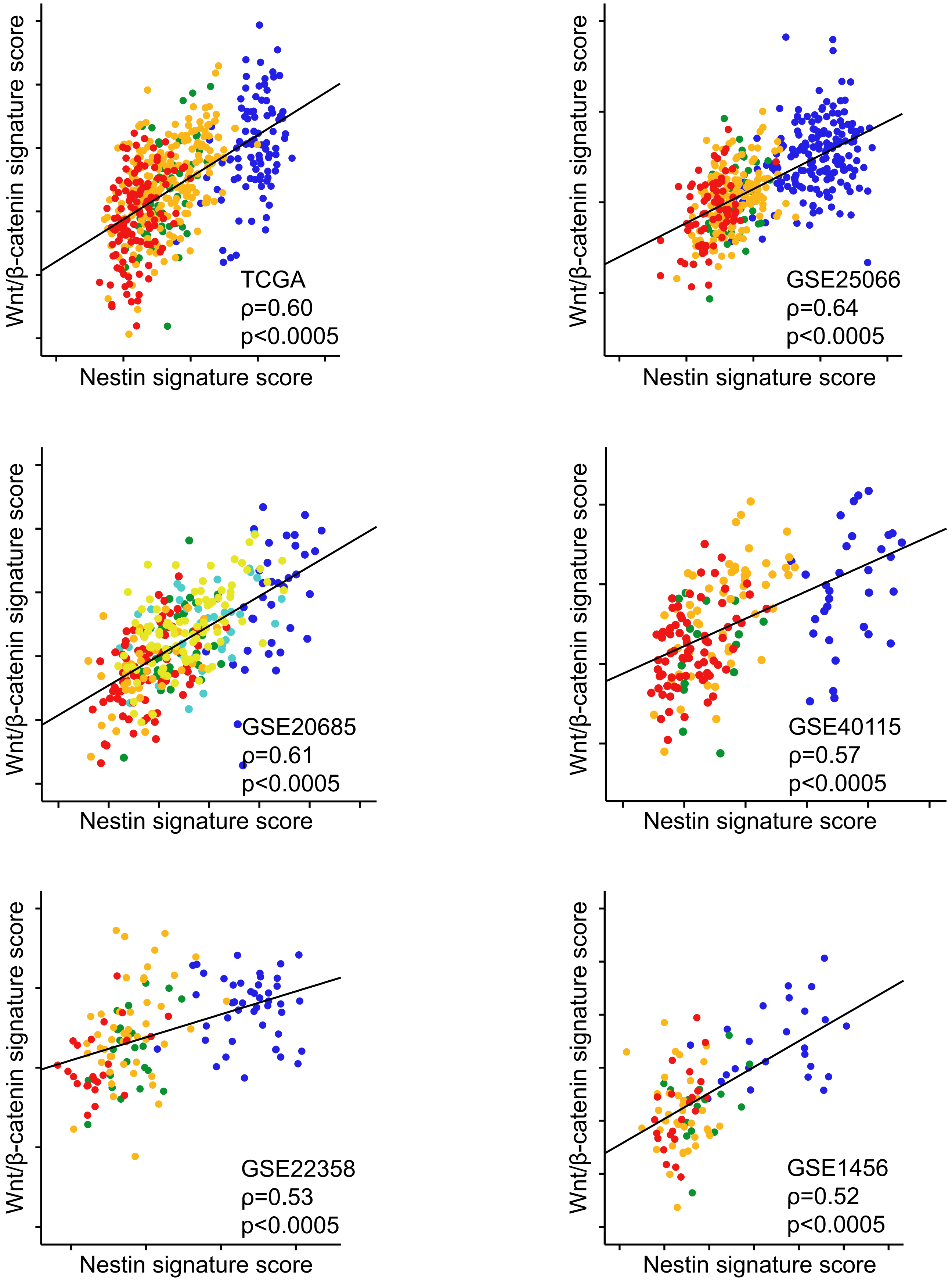
Wnt/β-catenin signature score
Nestin signature score
TCGA
ρ=0.60
p<0.0005
Wnt/β-catenin signature score
Nestin signature score
GSE25066
ρ=0.64
p<0.0005
Wnt/β-catenin signature score
Nestin signature score
GSE20685
ρ=0.61
p<0.0005
Wnt/β-catenin signature score
Nestin signature score
GSE40115
ρ=0.57
p<0.0005
Wnt/β-catenin signature score
Nestin signature score
GSE22358
ρ=0.53
p<0.0005
Wnt/β-catenin signature score
Nestin signature score
GSE1456
ρ=0.52
p<0.0005

Figure S4C

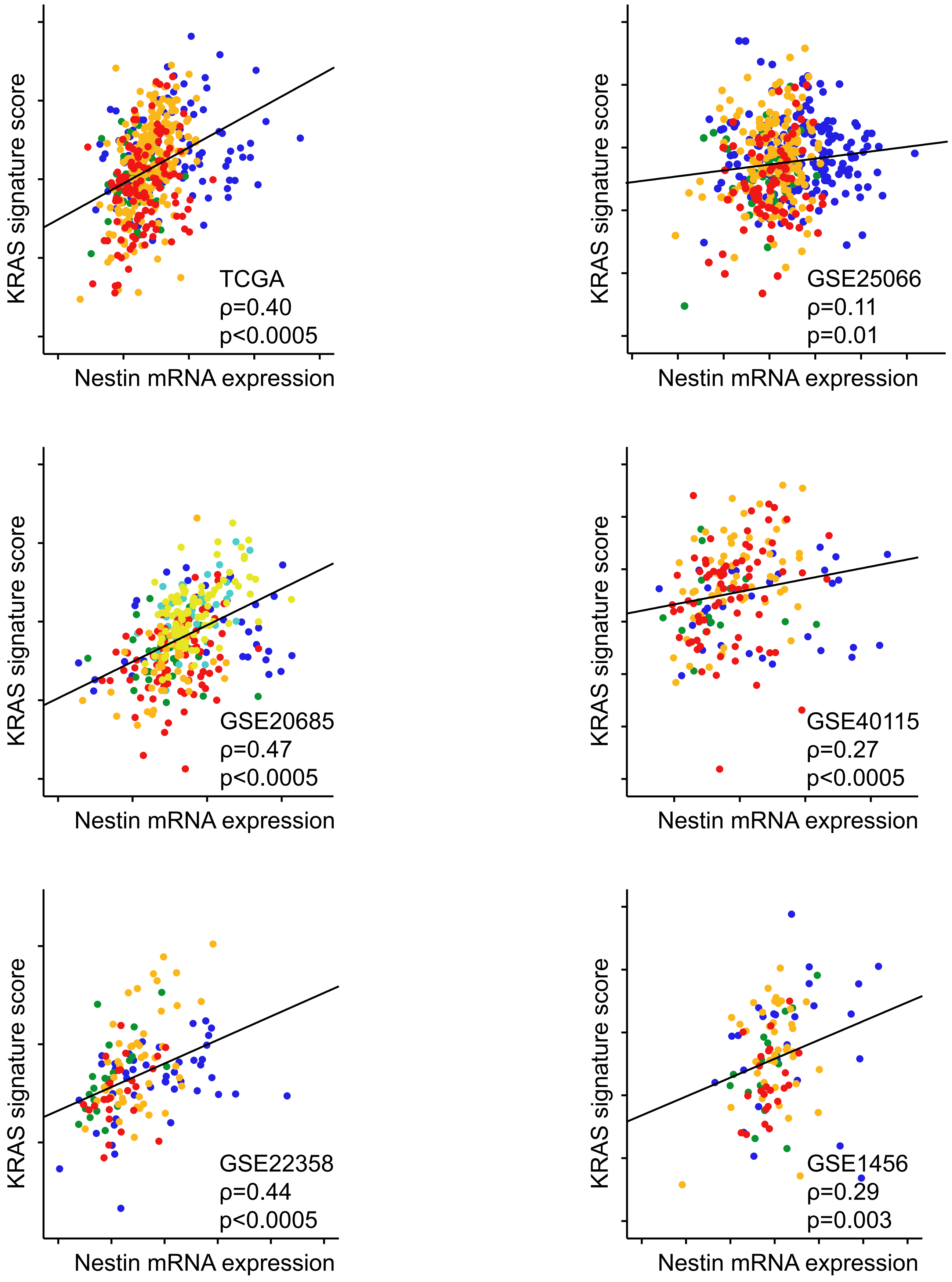
KRAS signature score
Nestin mRNA expression
TCGA
ρ=0.40
p<0.0005
GSE25066
ρ=0.11
p=0.01
GSE20685
ρ=0.47
p<0.0005
GSE40115
ρ=0.27
p<0.0005
GSE22358
ρ=0.44
p<0.0005
GSE1456
ρ=0.29
p=0.003

Figure S4D

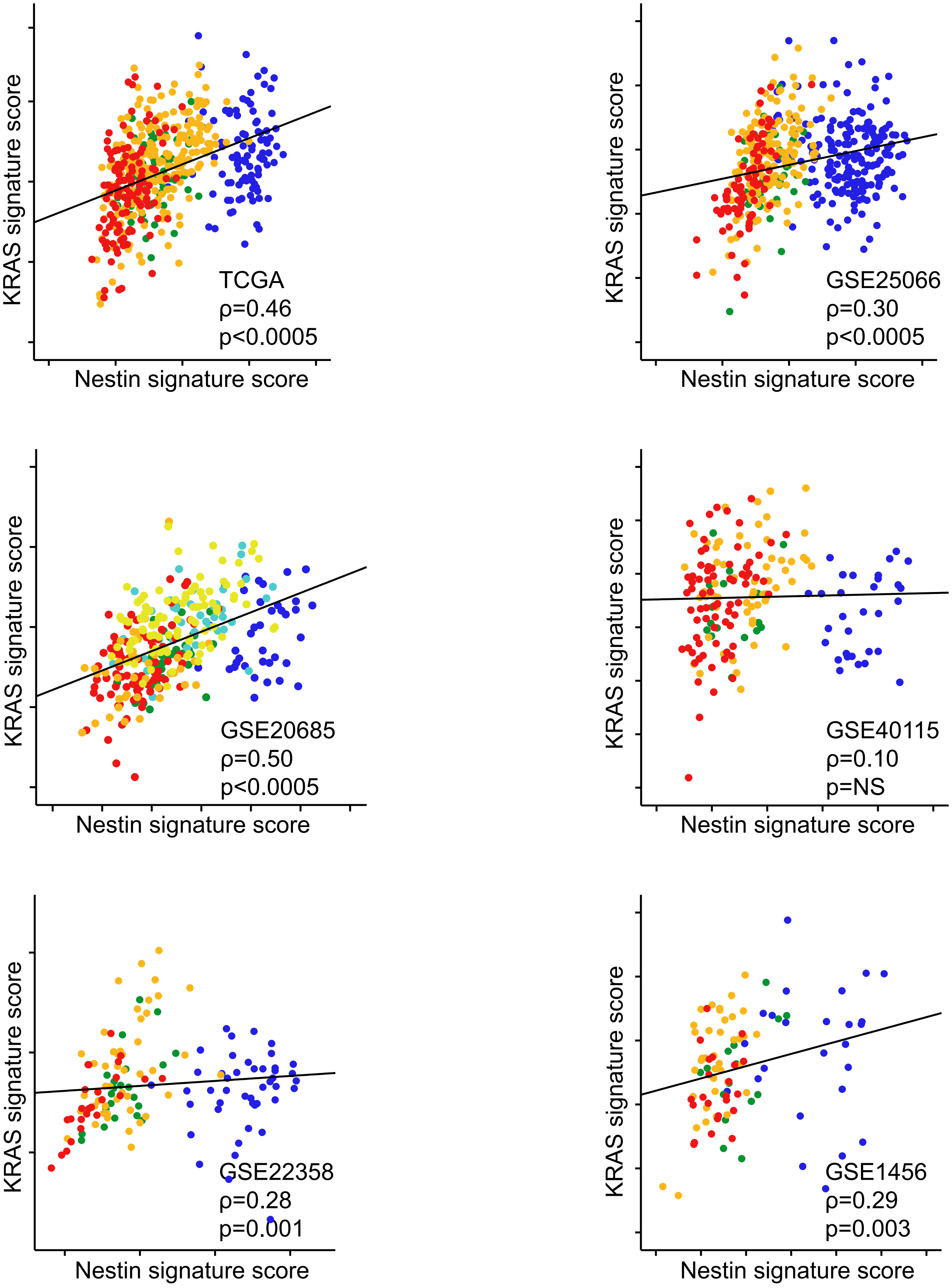
KRAS signature score
Nestin signature score
TCGA
ρ=0.46
p<0.0005
KRAS signature score
Nestin signature score
GSE25066
ρ=0.30
p<0.0005
KRAS signature score
Nestin signature score
GSE20685
ρ=0.50
p<0.0005
KRAS signature score
Nestin signature score
GSE40115
ρ=0.10
p=NS
KRAS signature score
Nestin signature score
GSE22358
ρ=0.28
p=0.001
KRAS signature score
Nestin signature score
GSE1456
ρ=0.29
p=0.003

Figure S5

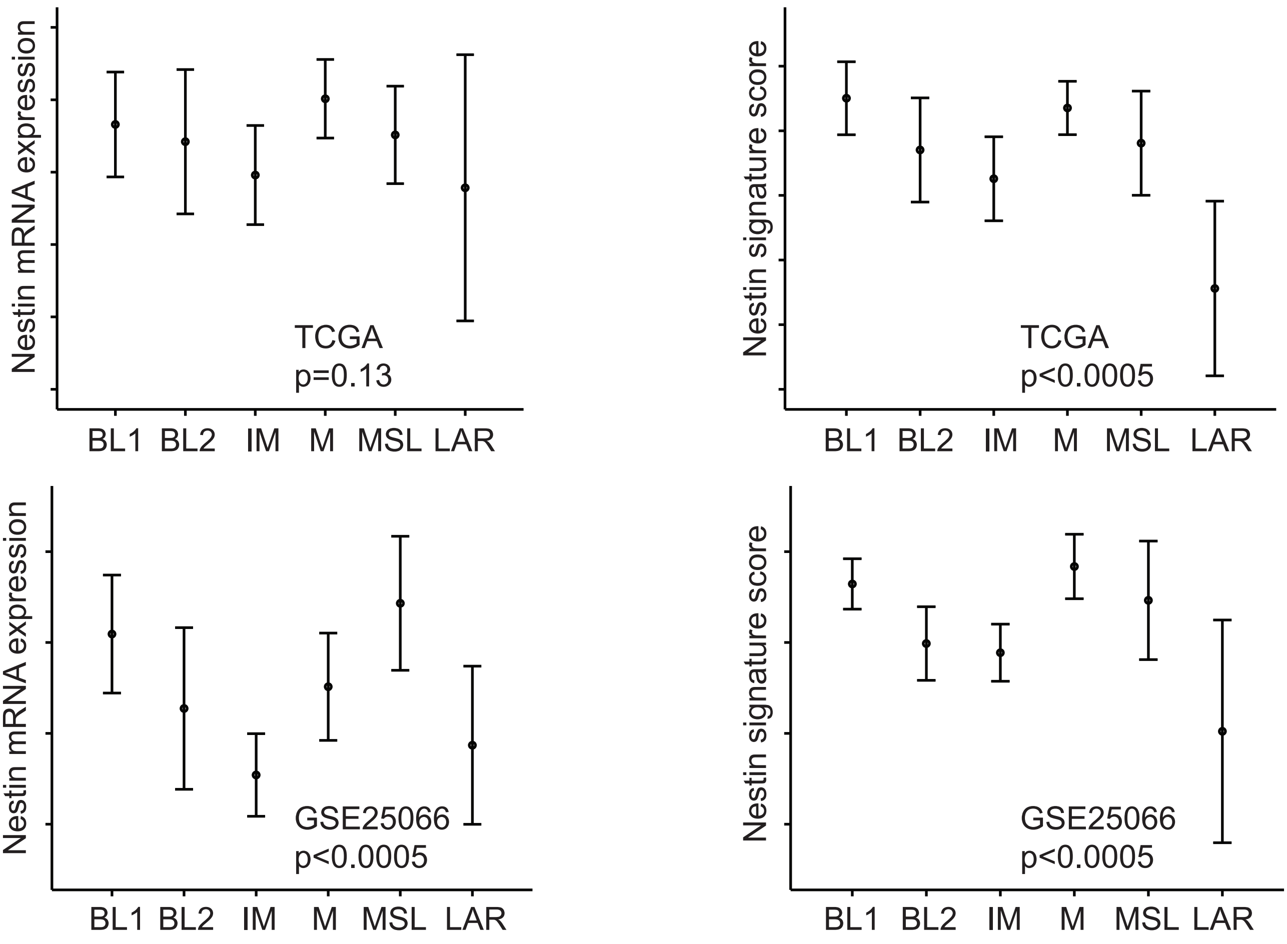
Nestin mRNA expression
TCGA
p=0.13
BL1 BL2 IM M MSL LAR
Nestin signature score
TCGA
p<0.0005
BL1 BL2 IM M MSL LAR
Nestin mRNA expression
GSE25066
p<0.0005
BL1 BL2 IM M MSL LAR
Nestin signature score
GSE25066
p<0.0005
BL1 BL2 IM M MSL LAR

Figure S6

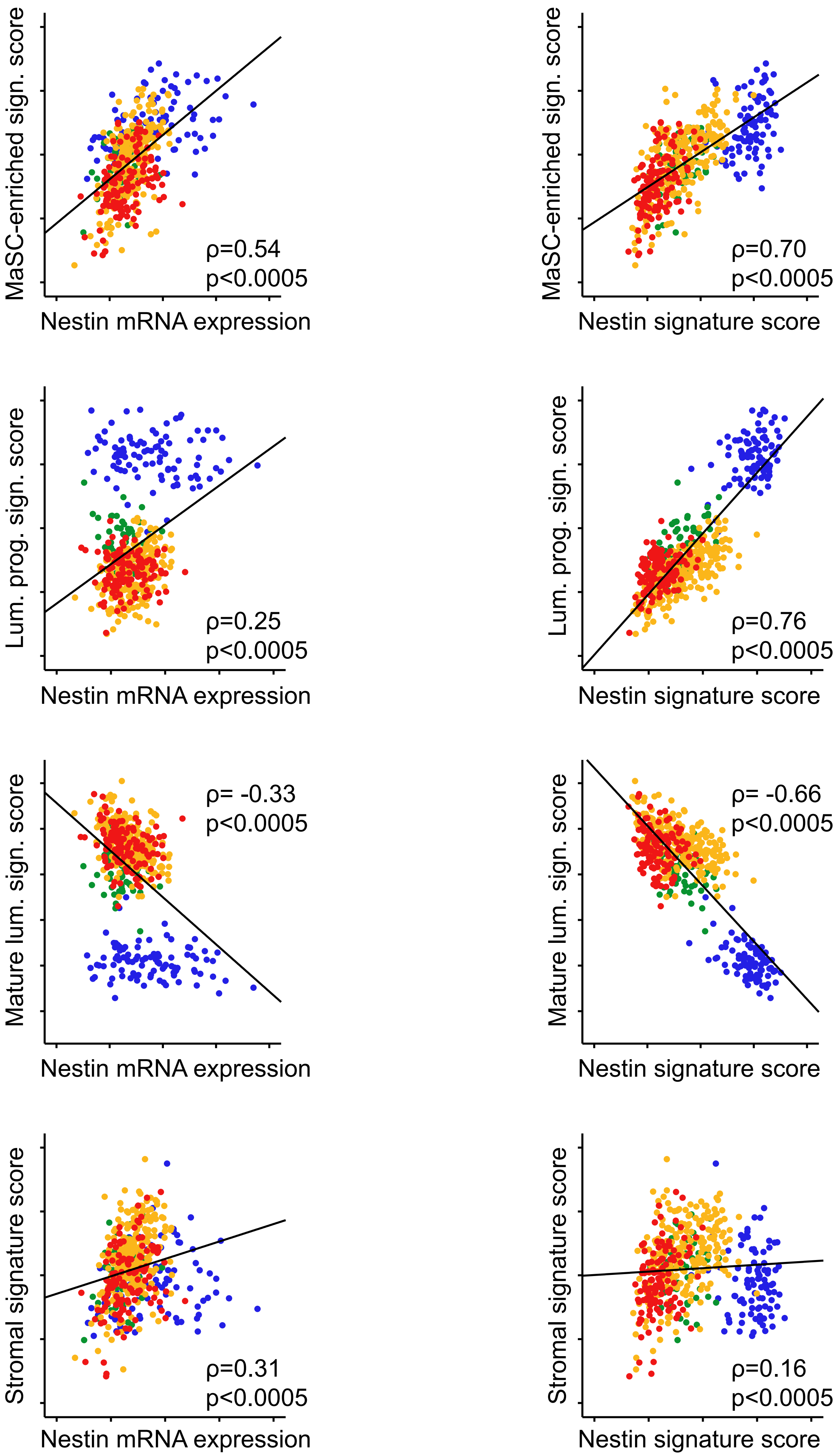
MaSC-enriched sign. score
ρ=0.54
p<0.0005
Nestin mRNA expression
MaSC-enriched sign. score
ρ=0.70
p<0.0005
Nestin signature score
Lum. prog. sign. score
ρ=0.25
p<0.0005
Nestin mRNA expression
Lum. prog. sign. score
ρ=0.76
p<0.0005
Nestin signature score
Mature lum. sign. score
ρ= -0.33
p<0.0005
Nestin mRNA expression
Mature lum. sign. score
ρ= -0.66
p<0.0005
Nestin signature score
Stromal signature score
ρ=0.31
p<0.0005
Nestin mRNA expression
Stromal signature score
ρ=0.16
p<0.0005
Nestin signature score

Table S1. Nestin protein expression and basal-like markers.

	Series I (n=528)			Series II (n=279)			Series III (n=181)			Series IV (n=187)		
**Variables**	**Nestin +**		**OR (95% CI)**	**Nestin +**		**OR (95% CI)**	**Nestin +**		**OR (95% CI)**	**Nestin +**		**OR (95% CI)**
	**n**	**(%)**		**n**	**(%)**		**n**	**(%)**		**n**	**(%)**	
CK5												
Negative	26	(5.7)	1.0	18	(7.6)	1.0	24	(17.1)	1.0	24	(15.4)	1.0
Positive	24	(35.3)	9.1 (4.8-17.2)***	17	(41.5)	8.7 (3.9-19.0)***	27	(65.9)	9.3 (4.3-20.4)***	20	(71.4)	13.8 (5.4-34.8)***
P-cadherin												
Negative	21	(5.0)	1.0	19	(7.9)	1.0	10	(10.1)	1.0	18	(13.2)	1.0
Positive	29	(26.9)	7.0 (3.8-12.8)***	16	(42.1)	8.5 (3.8-18.8)[a]***	41	(50.0)	8.9 (4.1-19.5)***	26	(53.1)	7.4 (3.5-15.7)***
EGFR												
Negative	14	(8.9)	1.0	-	-	-	36	(24.0)	1.0	23	(15.5)	1.0
Positive	4	(26.7)	3.7 (1.1-13.3)[a]	-	-	-	15	(53.6)	3.7 (1.6-8.4)**	21	(60.0)	8.2 (3.6-18.3)***
p53												
Low	9	(5.8)	1.0	-	-	-	26	(19.8)	1.0	24	(18.5)	1.0
High	11	(34.4)	8.5 (3.1-22.9)[a]***	-	-	-	25	(50.0)	4.0 (2.0-8.1)***	20	(36.4)	2.5 (1.2-5.1)**
BLP 1												
Absent	30	(6.1)	1.0	22	(8.4)	1.0	24	(16.3)	1.0	24	(15.3)	1.0
Present	20	(60.6)	23.8 (10.8-52.5)[a]***	13	(76.5)	35.5 (10.7-118)[a]***	27	(79.4)	19.8 (7.7-50.6)***	20	(74.1)	15.8 (6.0-41.5)***
BLP 2												
Absent	27	(5.5)	1.0	25	(9.7)	1.0	10	(8.2)	1.0	18	(12.4)	1.0
Present	23	(60.5)	26.3 (12.3-56.1)[a]***	10	(50.0)	9.4 (3.6-24.7)[a]***	41	(69.5)	25.5 (10.9-59.8)***	26	(65.0)	13.1 (5.8-29.6)***
BLP 3												
Absent	15	(8.6)	1.0	-	-	-	36	(22.5)	1.0	23	(14.9)	1.0
Present	4	(50.0)	10.7 (2.4-47.0)[a]**	-	-	-	15	(78.9)	12.9 (4.0-41.4)***	21	(70.0)	13.3 (5.4-32.6)***
BLP 4 (CBP)												
Absent	8	(4.8)	1.0	-	-	-	22	(15.6)	1.0	15	(10.4)	1.0
Present	11	(57.9)	27.0 (8.5-85.7)[a]***	-	-	-	29	(76.3)	17.4 (7.3-41.8)***	29	(72.5)	22.7 (9.4-54.4)***
BLP 5												
Present	7	(4.3)	1.0	-	-	-	9	(7.6)	1.0	12	(8.8)	1.0
Absent	13	(54.2)	26.2 (8.7-78.9)[a]***	-	-	-	42	(68.9)	26.8 (11.2-63.9)***	32	(66.7)	20.7 (8.9-48.0)***

[a] Fisher's exact test. n: number of patients; OR: odds ratio; CI: confidence interval; p-values by Pearson's chi-square test, [*] <0.05, [**] <0.01, [***] <0.001; CK: cytokeratin; EGFR: epidermal growth factor receptor; ER: oestrogen receptor; PR: progesterone receptor; HER2: human epidermal growth factor receptor 2; BLP; Basal-like profile; CBP: core basal phenotype. Cut-off values: ER and PR positive: ≥10%; HER2: see Supplementary Methods; CK5 positive: SI (staining index) >0; P-cadherin positive: SI >3; EGFR, positive: >1%, Dako criteria; p53 positive: SI >3. BLP 1-5; 1: ER- HER2- CK5+, 2: ER- HER2- P-cadherin+, 3: ER- HER2- EGFR+, 4: ER- HER2- CK5+ and/or EGFR+, 5: ER- HER2- CK5+ and/or P-cadherin+ and/or EGFR+. Missing data: Series I: CK5: n=1, P-cadherin: n=1, EGFR: n=355, p53: n=341, BLP 3: n=345, BLP 4: n=344, BLP 5: n=342, Series III: EGFR: n=3, BLP 3: n=2, BLP 4: n=2, BLP 5: n=2, Series IV: CK5: n=3, P-cadherin: n=2, EGFR: n=4, p53: n=2, BLP 1: n=3, BLP 2: n=2, BLP 3: n=3, BLP 4: n=3, BLP 5: n=3.

Table S2A. Basal-like subtype (n=95) predicted by gene expression values; multivariate logistic regression analysis (TCGA dataset, microarray data).

Variables	Patients, n	(%)	OR (95% CI)	*P*
Nestin	505	(100)	2.3 (1.6-3.5)	<0.0005
CK5	505	(100)	1.1 (0.9-1.4)	NS
EGFR	505	(100)	3.6 (2.5-5.1)	<0.0005
P-cadherin	505	(100)	2.6 (1.8-3.8)	<0.0005

n: number of patients; OR: odds ratio; CI: confidence interval; P: p-values. CK: cytokeratin; EGFR: epidermal growth factor receptor. Basal-like subtype was compared with luminal A, B, HER2 (human epidermal growth factor receptor 2) enriched subtypes. Normal breast-like cases were excluded. Nestin, CK5, EGFR, and P-cadherin were analysed as continuous variables.

Table S2B. Basal-like subtype (n=25) predicted by protein expression values; multivariate logistic regression analysis (TCGA dataset, proteomic data).

Variables	Patients, n	(%)	OR (95% CI)	*P*
Nestin	103	(100)	10.5 (2.9-37.3)	<0.0005
CK5	103	(100)	2.5 (1.2-5.1)	0.013
EGFR	103	(100)	4.0 (1.9-8.6)	<0.0005
P-cadherin	103	(100)	3.3 (1.1-9.9)	0.032

n: number of patients; OR: odds ratio; CI: confidence interval; P: p-values. CK: cytokeratin; EGFR: epidermal growth factor receptor. Basal-like subtype was compared with luminal A, B, HER2 (human epidermal growth factor receptor 2) enriched subtypes. Nestin, CK5, EGFR, and P-cadherin were analysed as continuous variables.

Table S3. Nestin protein expression by clinico-pathological and angiogenesis-related variables.

Variables	Series I (n=528)		Series II (n=279)		Series III (n=181)		Series IV (n=187)	
	Nestin + n (%)	OR (95% CI)	Nestin + n (%)	OR (95% CI)	Nestin + n (%)	OR (95% CI)	Nestin + n (%)	OR (95% CI)
Histological[a] type								
Ductal	39 (8.8)	1.0	30 (13.3)	1.0	45 (30.2)	-	37 (22.8)	-
Lobular	2 (3.7)	0.4 (0.1-1.7)[b]	4 (12.1)	0.9 (0.3-2.7)[b]	0 (0.0)	-[b*]	0 (0.0)	-[b]
Histological grade								
1-2	17 (3.9)	1.0	13 (6.0)	1.0	7 (7.9)	1.0	13 (14.6)	1.0
3	33 (36.7)	14.3 (7.5-27.4)***	22 (34.4)	8.1 (3.8-17.4)***	41 (51.2)	12.3 (5.1-29.9)***	31 (32.0)	2.7 (1.3-5.7)**
Tumour diameter								
< 2.0 cm	28 (7.8)	1.0	20 (9.8)	1.0	17 (21.0)	1.0	- -	-
≥ 2.0 cm	22 (13.0)	1.8 (1.0-3.2)	15 (20.3)	2.4 (1.1-4.9)*	32 (34.8)	2.0 (1.0-4.0)*	- -	-
Nodal status								
Negative	35 (9.2)	1.0	21 (11.4)	1.0	25 (41.7)	1.0	- -	-
Positive	15 (10.6)	1.2 (0.6-2.2)	14 (14.9)	1.4 (0.7-2.8)	9 (15.3)	0.3 (0.1-0.6)**	- -	-
Detection								
Screening	31 (8.1)	1.0	18 (9.1)	1.0	- -	-	- -	-
Interval	19 (13.3)	1.8 (1.0-3.2)	17 (21.0)	2.7 (1.3-5.5)**	- -	-	- -	-
VPI								
Low	11 (8.3)	1.0	- -	-	29 (22.3)	1.0	24 (18.9)	1.0
High	9 (20.5)	2.9 (1.1-7.4)[b]	- -	-	22 (46.8)	3.1 (1.5-6.2)**	19 (41.3)	3.0 (1.4-6.3)**
GMP								
Absent	8 (6.0)	1.0	- -	-	- -	-	- -	-
Present	12 (27.3)	5.9 (2.2-15.7)[b]***	- -	-	- -	-	- -	-
BVI								
Absent	14 (8.9)	1.0	25 (10.6)	1.0	- -	-	- -	-
Present	6 (20.7)	2.7 (0.9-7.7)[b]	10 (23.3)	2.6 (1.1-5.8)*	- -	-	- -	-
LVI								
Absent	16 (11.0)	1.0	20 (10.6)	1.0	- -	-	- -	-
Present	4 (9.5)	0.8 (0.3-2.7)[b]	15 (16.7)	1.7 (0.8-3.5)	- -	-	- -	-

[a] Cases with histological type other than ductal or lobular were not included in the analysis. [b] Fisher's exact test. n: number of patients; OR: odds ratio; CI: confidence interval; P-values by Pearson's chi-square test, * <0.05, ** <0.01, *** <0.001; VPI: vascular proliferation index; GMP: glomeruloid microvascular proliferation; BVI: blood vessel invasion; LVI: lymph vessel invasion. Cut-off values: VPI: series specific upper quartile was used; Series II: 5.44%, Series III: 2.25%, Series IV: 3.10%. Missing data: Series I: nodal status: n=5, VPI: n=351, GMP: n=350, BVI: n=341, LVI: n=341, Series II: nodal status: n=1, Series III: histological grade: n=12, tumour diameter: n=8, nodal status: n=62, VPI: n=4, Series IV: histological type: n=1, histological grade: n=1, VPI: n=14.

Table S4. Characteristics of the study populations, Series II, III, and IV are compared to Series I.

	Series I (n=546) n (%)	Series II (n=282) n (%)	*P*	Series III (n=202) n (%)	*P*	Series IV (n=192) n (%)	*P*
Age							
Years mean, range	59.9, 49 - 72	60.6, 49 - 70	0.07[a]	48.1, 19 - 82	<0.0005[a]	46.2, 18 - 80	<0.0005[a]
≤ 50 years	21 (3.8)	9 (3.2)		122 (62.6)		124 (67.4)	
> 50 years	525 (96.2)	273 (96.8)		73 (37.4)		60 (32.6)	
Histological[b] type							
Ductal	457 (83.7)	228 (80.9)		163 (87.2)		166 (86.9)	
Lobular	55 (10.1)	34 (12.1)	NS	15 (8.0)	NS	5 (2.6)	0.002
Histological grade							
1-2	456 (83.5)	218 (77.3)		100 (53.5)		93 (48.7)	
3	90 (16.5)	64 (22.7)	0.03	87 (46.5)	<0.0005	98 (51.3)	<0.0005
Tumour diameter							
< 2 cm	376 (68.9)	206 (73.0)		89 (46.4)		- -	
≥ 2 cm	170 (31.1)	76 (27.0)	NS	103 (53.6)	<0.0005	- -	-
Nodal status							
Negative	397 (73.5)	187 (66.5)		72 (53.7)		- -	
Positive	143 (26.5)	94 (33.5)	0.04	62 (46.3)	<0.0005	- -	-
ER							
Positive	462 (84.6)	247 (87.6)		100 (50.5)		72 (38.5)	
Negative	84 (15.4)	35 (12.4)	NS	98 (49.5)	<0.0005	115 (61.5)	<0.0005
PR							
Positive	389 (71.2)	186 (66.0)		98 (50.3)		53 (28.3)	
Negative	157 (28.8)	96 (34.0)	NS	97 (49.7)	<0.0005	134 (71.7)	<0.0005
HER2							
Negative	464 (86.7)	255 (90.4)		180 (90.9)		156 (83.4)	
Positive	71 (13.3)	27 (9.6)	NS	18 (9.1)	NS	31 (16.6)	NS
BRCA status (germline)							
0	- -	- -		104 (51.5)	-	- -	
1	- -	- -		53 (26.2)	-	- -	
2	- -	- -	-	45 (22.3)	-	- -	-

[a] Mann-Whitney U test. [b] Cases with histological type other than ductal or lobular were not included in the analysis. n: number of patients; *P*: p-values, by Pearson's chi-square test, Series II-IV are compared to Series I; NS: not significant; ER: oestrogen receptor; PR: progesterone receptor; HER2: human epidermal growth factor receptor 2. Cut-off values: ER and PR positive: ≥10%; HER2: see Supplementary Methods. Missing data: Series I: nodal status: n=6, HER2: n=11; Series II: nodal status: n=1, Series III: age: n=7, histological type: n=15, histological grade: n=15, tumour diameter: n=10, nodal status: n=68, ER: n=4, PR: n=7, HER2: n=4, Series IV: age: n=8, histological type: n=1, histological grade: n=1, ER: n=5, PR: n=5, HER2: n=5.

Table S5. Nestin gene expression signature (see methods, gene expression analyses)

Gene name	Entrez ID	Description	Fold change
**Upregulated**			
SFRP1	6422	secreted frizzled-related protein 1	5.6
ROPN1	54763	rhophilin associated tail protein 1	5.6
ROPN1B	152015	rhophilin associated tail protein 1B	5.4
MIA	8190	melanoma inhibitory activity	5.2
SOSTDC1	25928	sclerostin domain containing 1	4.8
KLK8	11202	kallikrein related peptidase 8	4.0
EDN3	1908	endothelin 3	3.5
GABRP	2568	gamma-aminobutyric acid type A receptor pi subunit	3.5
BBOX1	8424	gamma butyrobetaine hydroxylase 1	3.4
FABP7	2173	fatty acid binding protein 7	3.2
KLK6	5653	kallikrein related peptidase 6	3.1
CAPN6	827	calpain 6	3.1
PTCHD1	139411	patched domain containing 1	3.1
KRT6B	3854	keratin 6B, type II	3.0
S100B	6285	S100 calcium binding protein B	2.9
NES	10763	nestin	2.9
SHC4	399694	SHC (Src homology 2 domain containing) family member 4	2.9
KLK7	5650	kallikrein related peptidase 7	2.9
ELF5	2001	E74-like factor 5 (ets domain transcription factor)	2.8
LEMD1	93273	LEM domain containing 1	2.8
PROM1	8842	prominin 1	2.8
FOXC1	2296	forkhead box C1	2.8
MGC102966	644945	keratin 16 pseudogene 3 (KRT16P3)	2.8
KRT6A	3853	keratin 6A, type II	2.8
OSR1	130497	odd-skipped related transcription factor 1	2.8
KLK5	25818	kallikrein related peptidase 5	2.8
ID4	3400	inhibitor of DNA binding 4, dominant negative helix-loop-helix protein	2.8
DCX	1641	doublecortin	2.8
BCL11A	53335	B-cell CLL/lymphoma 11A	2.7
SCRG1	11341	stimulator of chondrogenesis 1	2.7
KRT6C	286887	keratin 6C, type II	2.7
PPP1R14C	81706	protein phosphatase 1 regulatory inhibitor subunit 14C	2.7
DSG3	1830	desmoglein 3	2.6
PTX3	5806	pentraxin 3	2.6
KRT17	3872	keratin 17, type I	2.6
STAC2	342667	SH3 and cysteine rich domain 2	2.6
hCG_1990170	285016	family with sequence similarity 150 member B (FAM150B)	2.6
KRT5	3852	keratin 5, type II	2.6
SOX10	6663	SRY-box 10	2.6
**Downregulated**			
AGR3	155465	anterior gradient 3, protein disulphide isomerase family member	-2.6
C1orf64	149563	chromosome 1 open reading frame 64	-2.8
AGR2	10551	anterior gradient 2, protein disulphide isomerase family member	-2.9
FOXA1	3169	forkhead box A1	-3.3
ESR1	2099	oestrogen receptor 1	-3.3

REFERENCES

1 Knutsvik, G. *et al.* Evaluation of Ki67 expression across distinct categories of breast cancer specimens: a population-based study of matched surgical specimens, core needle biopsies and tissue microarrays. *PLoS One.* **9,** e112121 (2014).

2 Klingen, T. A. *et al.* Tumour cell invasion into blood vessels is significantly related to breast cancer subtypes and decreased survival. *J Clin Pathol.* (2016).

3 Wabinga, H. R., Parkin, D. M., Wabwire-Mangen, F. & Mugerwa, J. W. Cancer in Kampala, Uganda, in 1989-91: changes in incidence in the era of AIDS. *Int J Cancer.* **54,** 26-36 (1993).

4 Nalwoga, H., Arnes, J. B., Wabinga, H. & Akslen, L. A. Expression of aldehyde dehydrogenase 1 (ALDH1) is associated with basal-like markers and features of aggressive tumours in African breast cancer. *Br J Cancer.* **102,** 369-375 (2010).

5 Gjerdrum, C. *et al.* Axl is an essential epithelial-to-mesenchymal transition-induced regulator of breast cancer metastasis and patient survival. *Proc Natl Acad Sci U S A.* **107,** 1124-1129 (2010).

6 Zhu, Y., Qiu, P. & Ji, Y. TCGA-assembler: open-source software for retrieving and processing TCGA data. *Nat Methods.* **11,** 599-600 (2014).

7 Parker, J. S. *et al.* Supervised risk predictor of breast cancer based on intrinsic subtypes. *J Clin Oncol.* **27,** 1160-1167 (2009).

8 Cancer Genome Atlas, N. Comprehensive molecular portraits of human breast tumours. *Nature.* **490,** 61-70 (2012).

9 Curtis, C. *et al.* The genomic and transcriptomic architecture of 2,000 breast tumours reveals novel subgroups. *Nature.* **486,** 346-352 (2012).

10 Hatzis, C. *et al.* A genomic predictor of response and survival following taxane-anthracycline chemotherapy for invasive breast cancer. *JAMA.* **305,** 1873-1881 (2011).

11 Itoh, M. *et al.* Estrogen receptor (ER) mRNA expression and molecular subtype distribution in ER-negative/progesterone receptor-positive breast cancers. *Breast Cancer Res Treat.* **143,** 403-409 (2014).

12 Kao, K. J., Chang, K. M., Hsu, H. C. & Huang, A. T. Correlation of microarray-based breast cancer molecular subtypes and clinical outcomes: implications for treatment optimization. *BMC Cancer.* **11,** 143 (2011).

13 Larsen, M. J. *et al.* Classifications within molecular subtypes enables identification of BRCA1/BRCA2 mutation carriers by RNA tumor profiling. *PLoS One.* **8,** e64268 (2013).

14 Gluck, S. *et al.* TP53 genomics predict higher clinical and pathologic tumor response in operable early-stage breast cancer treated with docetaxel-capecitabine +/- trastuzumab. *Breast Cancer Res Treat.* **132,** 781-791 (2012).

15 Pawitan, Y. *et al.* Gene expression profiling spares early breast cancer patients from adjuvant therapy: derived and validated in two population-based cohorts. *Breast Cancer Res.* **7,** R953-964 (2005).

16 Jonsson, G. *et al.* The retinoblastoma gene undergoes rearrangements in BRCA1-deficient basal-like breast cancer. *Cancer Res.* **72,** 4028-4036 (2012).

17 Perou, C. M. *et al.* Molecular portraits of human breast tumours. *Nature.* **406,** 747-752 (2000).

18 Sorlie, T. *et al.* Gene expression patterns of breast carcinomas distinguish tumor subclasses with clinical implications. *Proc Natl Acad Sci U S A.* **98,** 10869-10874 (2001).

19 Gyorffy, B. *et al.* An online survival analysis tool to rapidly assess the effect of 22,277 genes on breast cancer prognosis using microarray data of 1,809 patients. *Breast Cancer Res Treat.* **123,** 725-731 (2010).

20 Foulkes, W. D. *et al.* The prognostic implication of the basal-like (cyclin E high/p27 low/p53+/glomeruloid-microvascular-proliferation+) phenotype of BRCA1-related breast cancer. *Cancer Res.* **64,** 830-835 (2004).

21 Chappuis, P. O. *et al.* Germline BRCA1/2 mutations and p27(Kip1) protein levels independently predict outcome after breast cancer. *J Clin Oncol.* **18,** 4045-4052 (2000).

22 Prat, A. *et al.* Molecular features of the basal-like breast cancer subtype based on BRCA1 mutation status. *Breast Cancer Res Treat.* **147,** 185-191 (2014).

23 Won, J. R. *et al.* A survey of immunohistochemical biomarkers for basal-like breast cancer against a gene expression profile gold standard. *Mod Pathol.* **26,** 1438-1450 (2013).

24 Liu, C. *et al.* Clinical implications for nestin protein expression in breast cancer. *Cancer Sci.* **101,** 815-819 (2010).

25 Li, H. *et al.* Nestin is expressed in the basal/myoepithelial layer of the mammary gland and is a selective marker of basal epithelial breast tumors. *Cancer Res.* **67,** 501-510 (2007).

26 Collett, K. *et al.* A basal epithelial phenotype is more frequent in interval breast cancers compared with screen detected tumors. *Cancer Epidemiol Biomarkers Prev.* **14,** 1108-1112 (2005).

27 Collett, K. *et al.* Expression of enhancer of zeste homologue 2 is significantly associated with increased tumor cell proliferation and is a marker of aggressive breast cancer. *Clin Cancer Res.* **12,** 1168-1174 (2006).

28 Arnes, J. B., Collett, K. & Akslen, L. A. Independent prognostic value of the basal-like phenotype of breast cancer and associations with EGFR and candidate stem cell marker BMI-1. *Histopathology.* **52,** 370-380 (2008).

29 Chen, Y. *et al.* Breast cancer stromal elastosis is associated with mammography screening detection, low Ki67 expression and favourable prognosis in a population-based study. *Diagn Pathol.* **9,** 230 (2014).

30 Nalwoga, H. *et al.* Vascular proliferation is increased in basal-like breast cancer. *Breast Cancer Res Treat.* **130,** 1063-1071 (2011).

31 Goffin, J. R. *et al.* Glomeruloid microvascular proliferation is associated with p53 expression, germline BRCA1 mutations and an adverse outcome following breast cancer. *Br J Cancer.* **89,** 1031-1034 (2003).

32 Arnes, J. B. *et al.* Placental cadherin and the basal epithelial phenotype of BRCA1-related breast cancer. *Clin Cancer Res.* **11,** 4003-4011 (2005).

33 Arnes, J. B. *et al.* Vascular proliferation is a prognostic factor in breast cancer. *Breast Cancer Res Treat.* **133,** 501-510 (2012).

34 Foulkes, W. D. *et al.* Germline BRCA1 mutations and a basal epithelial phenotype in breast cancer. *J Natl Cancer Inst.* **95,** 1482-1485 (2003).

35 Arnes, J. B. *et al.* Expression of epidermal growth factor receptor in relation to BRCA1 status, basal-like markers and prognosis in breast cancer. *J Clin Pathol.* **62,** 139-146 (2009).

36 Nalwoga, H., Arnes, J. B., Wabinga, H. & Akslen, L. A. Frequency of the basal-like phenotype in African breast cancer. *APMIS.* **115,** 1391-1399 (2007).

37 Karp, S. E. *et al.* Influence of BRCA1 mutations on nuclear grade and estrogen receptor status of breast carcinoma in Ashkenazi Jewish women. *Cancer.* **80,** 435-441 (1997).

38 Rhodes, A., Jasani, B., Anderson, E., Dodson, A. R. & Balaton, A. J. Evaluation of HER-2/neu immunohistochemical assay sensitivity and scoring on formalin-fixed and paraffin-processed cell lines and breast tumors: a comparative study involving results from laboratories in 21 countries. *Am J Clin Pathol.* **118,** 408-417 (2002).

39 Weidner, N., Moore, D. H., 2nd & Vartanian, R. Correlation of Ki-67 antigen expression with mitotic figure index and tumor grade in breast carcinomas using the novel "paraffin"-reactive MIB1 antibody. *Hum Pathol.* **25,** 337-342 (1994).

40 Nalwoga, H., Arnes, J. B., Wabinga, H. & Akslen, L. A. Expression of EGFR and c-kit is associated with the basal-like phenotype in breast carcinomas of African women. *APMIS.* **116,** 515-525 (2008).

41 Straume, O. & Akslen, L. A. Alterations and prognostic significance of p16 and p53 protein expression in subgroups of cutaneous melanoma. *Int J Cancer.* **74,** 535-539 (1997).

42 Nielsen, T. O. *et al.* Immunohistochemical and clinical characterization of the basal-like subtype of invasive breast carcinoma. *Clin Cancer Res.* **10,** 5367-5374 (2004).

43 Stefansson, I. M., Salvesen, H. B. & Akslen, L. A. Vascular proliferation is important for clinical progress of endometrial cancer. *Cancer Res.* **66,** 3303-3309 (2006).

44 Kruger, K. *et al.* Microvessel proliferation by co-expression of endothelial nestin and Ki-67 is associated with a basal-like phenotype and aggressive features in breast cancer. *Breast.* **22,** 282-288 (2013).

45 Tusher, V. G., Tibshirani, R. & Chu, G. Significance analysis of microarrays applied to the ionizing radiation response. *Proc Natl Acad Sci U S A.* **98,** 5116-5121 (2001).

46 Subramanian, A. *et al.* Gene set enrichment analysis: a knowledge-based approach for interpreting genome-wide expression profiles. *Proc Natl Acad Sci U S A.* **102,** 15545-15550 (2005).

47 Stavrum, A. K., Petersen, K., Jonassen, I. & Dysvik, B. Analysis of gene-expression data using J-Express. *Curr Protoc Bioinformatics.* **Chapter 7,** Unit 7 3 (2008).

48 Dysvik, B. & Jonassen, I. J-Express: exploring gene expression data using Java. *Bioinformatics.* **17,** 369-370 (2001).

49 Lim, E. *et al.* Aberrant luminal progenitors as the candidate target population for basal tumor development in BRCA1 mutation carriers. *Nat Med.* **15,** 907-913 (2009).

50 Judeh, T., Johnson, C., Kumar, A. & Zhu, D. TEAK: topology enrichment analysis framework for detecting activated biological subpathways. *Nucleic Acids Res.* **41,** 1425-1437 (2013).

51 Kanehisa, M. & Goto, S. KEGG: kyoto encyclopedia of genes and genomes. *Nucleic Acids Res.* **28,** 27-30 (2000).

52 Lehmann, B. D. *et al.* Identification of human triple-negative breast cancer subtypes and preclinical models for selection of targeted therapies. *J Clin Invest.* **121,** 2750-2767 (2011).

53 Chen, X. *et al.* TNBCtype: A Subtyping Tool for Triple-Negative Breast Cancer. *Cancer Inform.* **11,** 147-156 (2012).

54 Bo, T. H., Dysvik, B. & Jonassen, I. LSimpute: accurate estimation of missing values in microarray data with least squares methods. *Nucleic Acids Res.* **32,** e34 (2004).

55 Li, Q., Birkbak, N. J., Gyorffy, B., Szallasi, Z. & Eklund, A. C. Jetset: selecting the optimal microarray probe set to represent a gene. *BMC Bioinformatics.* **12,** 474 (2011).
